# Optimizing dark fermentation for hydrogen production: lessons from *Thermoactinomyces mirandus*

**DOI:** 10.3389/fmicb.2026.1827551

**Published:** 2026-06-26

**Authors:** Selina V. Haller, Luise Ebert, Mira Mutschlechner, Harald Schöbel, Andreas O. Wagner

**Affiliations:** 1 Department Microbiology, Universität Innsbruck, Innsbruck, Austria; 2 Department Biotechnology, MCI Internationale Hochschule GmbH, Innsbruck, Austria

**Keywords:** biosynthesis, carbon-to-nitrogen ratio, competing pathways, inhibitory factors, lactose metabolism, pH control, process optimization, substrate utilization

## Abstract

Biological hydrogen (H_2_) production *via* dark fermentation (DF) offers a renewable pathway for energy recovery from biomass, but process performance is highly strain dependent and often limited by suboptimal operating conditions. The present study characterizes fermentation physiology and optimizes small-scale H_2_ production by the recently discovered thermophilic anaerobe *Thermoactinomyces mirandus*. Batch cultivations at 52 °C in anoxic serum bottles were used to screen carbon sources (glucose, fructose, xylose, arabinose, and lactose), nitrogen sources (yeast extract, casein peptone, casamino acids, and ammonium chloride), carbon to nitrogen (C/N) ratios, and a targeted set of potential inhibitory factors. Yeast extract supported the highest H_2_ yields, while fructose, xylose, and lactose were the most effective carbon sources. Baseline lactose fermentation without pH control yielded 118.31 ± 19.91 mmol H_2_ mol hexose equivalent^−1^ after 11 days, with incomplete lactose conversion. Implementing a small-scale, closed-flask pH control at setpoint 7.2 increased the yield 4.2-fold to 466.6 ± 10.2 mmol H_2_ mol hexose equivalent^−1^ and achieved 99.4 ± 0.2% lactose consumption. Daily nitrogen (N_2_) gas sparging modestly improved conversion, while 10-fold supplementation of trace elements, ferrous iron, or formate had no effect. Under controlled pH, an optimum pH range of 7.5–8.0 for maximum H_2_ production rate (VHPR) was identified. Higher lactose loads increased the VHPR by 147% from 5.6 to 13.9 mmol lactose L^−1^, with a trade-off in yield (26% decrease). Fermentation product profiles shifted under pH control, with ethanol and acetate increasing relative to lactate, consistent with enzyme pH optima and upregulation of *pflB* (pyruvate formate lyase) after 4 days cultivation. These results demonstrate that pH control is a key factor for enhancing H_2_ yield by *T. mirandus*, define quantitative operating windows, and deliver a transferable workflow to identify and mitigate inhibitory factors in DF processes.

## 1 Introduction

The global energy crisis and the environmental impacts of fossil fuel use have intensified efforts to develop sustainable and renewable energy systems (Cao et al., 2022; Dari et al., 2024; Łukajtis et al., 2018; Zhang et al., 2024). Hydrogen (H_2_) is a promising energy carrier due to its high specific energy, clean combustion, and cross-sector applicability in fuel cells, industry, and transportation (Beschkov et al., 2023; Dari et al., 2024; Evro et al., 2024). However, conventional H_2_ production [e.g., steam methane (CH_4_) reforming, water electrolysis] remains either dependent on non-renewable resources or energy intensive, constraining its environmental and economic sustainability (Dari et al., 2024; Evro et al., 2024). These limitations motivate interest in biological H_2_ production, particularly dark fermentation (DF), as a renewable and potentially cost-effective alternative (Cao et al., 2022; Dari et al., 2024; Dzulkarnain et al., 2022).

DF is an anaerobic microbial process that converts organic substrates into H_2_ and organic acids/alcohols without light (Ahmad et al., 2024; Ivanenko et al., 2024). It can valorize diverse feedstocks, including agricultural residues, industrial waste streams, and other biomass sources, thereby supporting resource recovery and waste minimization (Ahmad et al., 2024; Dari et al., 2024; Mohanakrishna et al., 2023). DF is performed by phylogenetically diverse bacteria, including the genera *Clostridium* (Wang and Yin, 2021), *Enterobacter* (Khanna et al., 2011; Kumar and Das, 2000) and *Thermoanaerobacterium* (Cao et al., 2014; Zhao et al., 2019).

**Figure 1:**
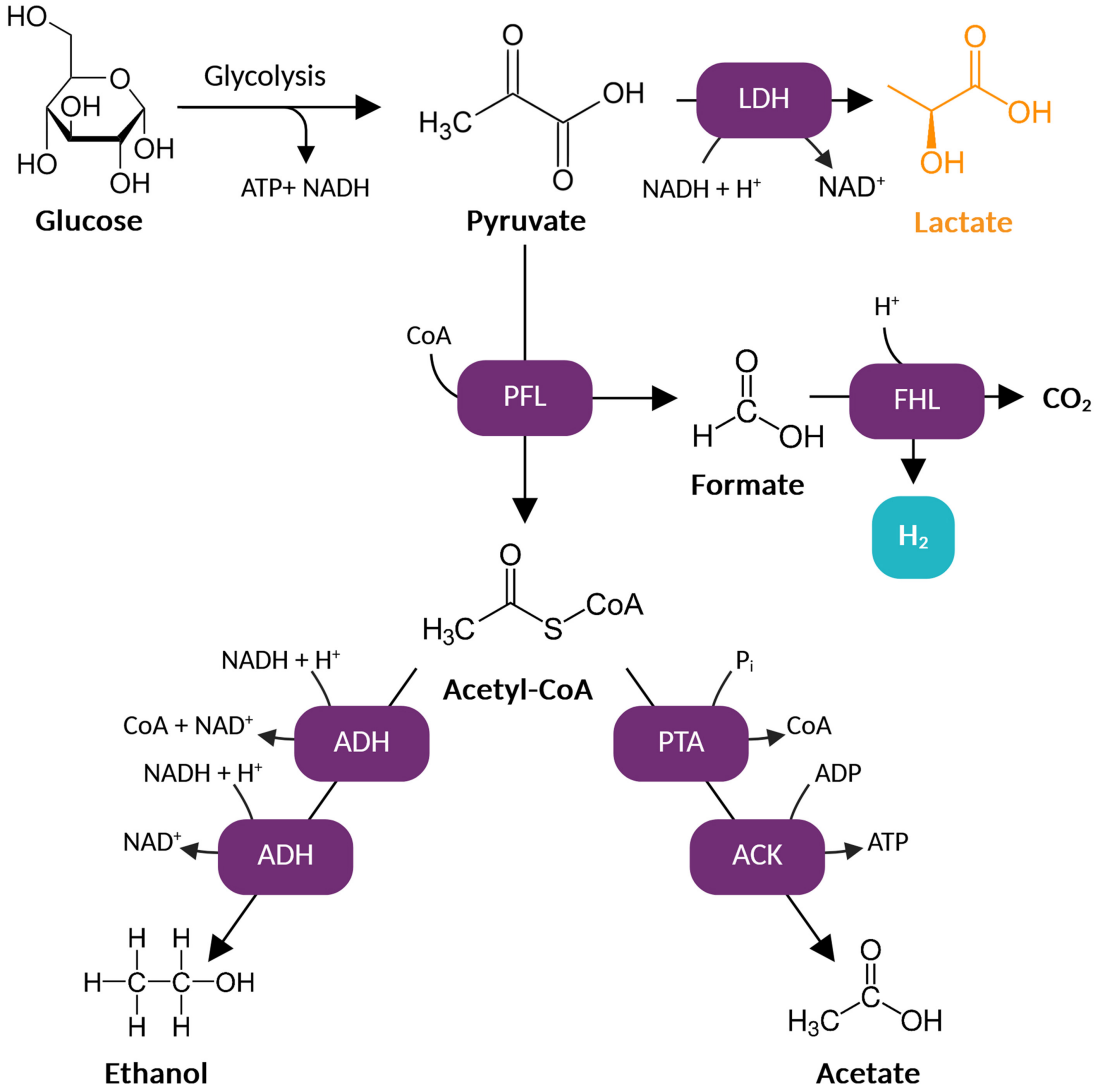
Overview of the FHL-dependent DF pathway from glucose. Glucose is metabolized to pyruvate *via* glycolysis. Per glucose molecule, each two molecules pyruvate, NADH and ATP are generated. Pyruvate is cleaved to acetyl-CoA and formate by PFL. Formate is oxidized to CO_2_ and H_2_ by the FHL complex containing a [NiFe]-hydrogenase, while acetyl-CoA is converted to ethanol and acetate *via* aldehyde/alcohol dehydrogenase (ADH), phosphate acetyltransferase (PTA), and acetate kinase (ACK). A competing branch diverts pyruvate to lactate *via* LDH. Figure modified after Vivijs et al. (2015) and Cao et al. (2022).

In facultative anaerobes, such as *Escherichia coli* and *Enterobacter aerogenes*, pyruvate is typically cleaved to acetyl-CoA and formate by pyruvate formate lyase (PFL), and formate is subsequently converted to H_2_ and carbon dioxide (CO_2_) by the formate hydrogen lyase (FHL) complex (Figure 1; Cao et al., 2022; Mathews et al., 2010; Zhao et al., 2019). When acetate and ethanol are the only by-products, this pathway yields a theoretical maximum of 2 mol H_2_ mol hexose equivalent^−1^ (Cao et al., 2022), while diversion of pyruvate to lactate via lactate dehydrogenase (LDH) reduces H_2_ yields (Hallenbeck, 2005).

DF performance depends on substrate composition, microbial strain, and operational parameters such as pH, temperature, gas-phase composition, and accumulation of fermentation products, rendering identification of inhibitory factors crucial for process control (Chen et al., 2021; Soares et al., 2020). Thermophilic conditions are frequently favored over mesophilic conditions due to enhanced substrate hydrolysis and improved contamination control (Mattiello-Francisco et al., 2024; Sillero et al., 2023).

Moreover, discovery of novel strains remains pivotal to broaden substrate flexibility and tolerance to process conditions (Mokhtarani et al., 2025; Rani et al., 2024; Vidal et al., 2025). *Thermoactinomyces mirandus* is a recently described thermophilic, filamentous anaerobic bacterium isolated from a biogas plant in Tyrol, Austria (Mutschlechner et al., 2021). It is the first anaerobic member of its genus and forms white, branching, septate mycelium that aggregates into pellets in liquid culture (Mutschlechner et al., 2021). The strain grows between 45 and 60 °C and at pH 5.0–9.0, and during lactose fermentation produces lactate, acetate, ethanol, CO_2_, and H_2_ via a [NiFe]-hydrogenase embedded in an FHL complex (Mutschlechner et al., 2021). To date, the organisms' substrate spectrum and conditions for optimal H_2_ production remain uncharacterized. Thus, the aims of the present study were to (i) characterize its fermentation physiology; (ii) screen degradable carbon sources and compatible nitrogen sources; and (iii) optimize small-scale H_2_ production by varying substrate concentrations, carbon-to-nitrogen (C/N) ratios, and pH. We also sought to identify and mitigate factors that impede complete substrate conversion to improve H_2_ yields. These results advance understanding of *T. mirandus* and provide a transferable workflow for diagnosing inhibitory factors and optimizing DF in other H_2_-producing strains.

## 2 Material and methods

### 2.1 Strain and culture conditions

*Thermoactinomyces mirandus* DSM 110094, originally isolated from a biogas plant in Roppen, Tyrol, Austria (Mutschlechner et al., 2021), was used as the H_2_-producing organism in the present investigation.

Batch cultivations were performed in 120 mL serum bottles with a 50 mL working volume. The basal medium was prepared following established protocols (Mutschlechner et al., 2021; Wagner et al., 2019) and contained, per liter: 0.35 g K_2_HPO_4_, 0.23 g KH_2_PO_4_, 0.5 g MgSO_4_ · 7 H_2_O, 0.05 g CaCl_2_ · 2 H_2_O, 2.25 g NaCl, 0.002 g FeSO_4_ · 7 H_2_O, 0.5 g sodium acetate, and 0.5 g L-cysteine HCl. After dissolving these components, 5 mL NaHCO_3_ stock solution (50 g L^−1^), 1 mL resazurin stock solution (0.1% w/v), and 1 mL SL-10 trace element solution (DSMZ Medium 120) were added. SL-10 contained 10 mL L^−1^ 7.7 M HCl, and per liter: 1500 mg FeCl_2_ · 4 H_2_O, 70 mg ZnCl_2_, 100 mg MnCl_2_ · 4 H_2_O, 6 mg H_3_BO_3_, 190 mg CoCl_2_ · 6 H_2_O, 2 mg CuCl_2_ · 2 H_2_O, 24 mg NiCl_2_ · 6 H_2_O, and 36 mg N_2_MoO_4_ · 2 H_2_O. The pH was adjusted to 6.5 ± 0.1 at room temperature using NaOH.

Autoclaved medium was dispensed into serum bottles under a nitrogen gas (N_2_) atmosphere to maintain anoxic conditions; these were immediately sealed with butyl rubber stoppers and aluminum crimp caps. To further reduce redox potential, 0.5 mL Na_2_S·9 H_2_O stock solution (12 g L^−1^) was added per bottle. Carbon and nitrogen source stock solutions were then added to achieve concentrations of 66.6 mmol L^−1^ carbon equivalents and 2.9 mmol L^−1^ nitrogen equivalents. This corresponds to C/N = 20. Unless noted otherwise, lactose monohydrate served as the carbon source and yeast extract as the nitrogen source. The initial carbon source concentration was confirmed by HPLC analysis and used for H_2_ yield calculations. The final pH of the medium was 7.2 ± 0.1.

Long-term preservation of the strain *T. mirandus* followed a protocol for deep-freezing of thermophilic microorganisms (Spring, 2006). Active, four-day old, *T. mirandus* cultures were concentrated and resuspended 1:1 in 5% sterile DMSO, frozen, and stored in the vapor phase of liquid N_2_.

Precultures were inoculated from the preserved cryocultures in basal medium and incubated at 52 °C for 4 days. For experimental inoculation, 2% v/v homogeneous preculture was transferred into each serum bottle. Headspace pressure was equilibrated to ambient pressure with N_2_ gas, and cultures were incubated statically at 52 °C for 8 days. For inoculation reproducibility, a triplicate of control serum bottles containing basal medium was run in parallel with every experiment. The average H_2_ production of all control flasks used in this study amounted to 1.2 ± 0.2 mmol L^−1^ (*n* = 30). Experiments with control flasks outside this range were excluded.

All tests were conducted in triplicates.

### 2.2 Experimental setup

The ensuing subsections illustrate the initial fermentation performance evaluation, subsequently accompanied by a systematic stepwise procedure to investigate and optimize H_2_ production by *T. mirandus*. First, carbon and nitrogen sources along with C/N ratios were screened to define nutrient requirements. Second, potential inhibiting factors were assessed and mitigated. Finally, the controlled pH was optimized and tested at higher carbon loads to assess performance under increased substrate supply.

#### 2.2.1 Evaluation of carbon and nitrogen source combinations and ratios

To screen carbon and nitrogen source combinations that support H_2_ production by *T. mirandus*, the basal medium was supplemented with a single carbon source comprising: glucose, fructose, xylose, arabinose, or lactose. Each carbon source was combined with a single nitrogen source including yeast extract, casein peptone or tryptone, Bacto casamino acids, or ammonium chloride.

In a subsequent experiment, the initial carbon concentrations were set to 66.6, 166.5, or 333.1 mmol L^−1^ on a molar basis of the carbon sources (fructose, xylose, or lactose). Yeast extract was supplied to achieve C/N = 5, 20, 50, 100, or 200 according to the supplier specifications.

#### 2.2.2 Identification of inhibiting factors

**Figure 2:**
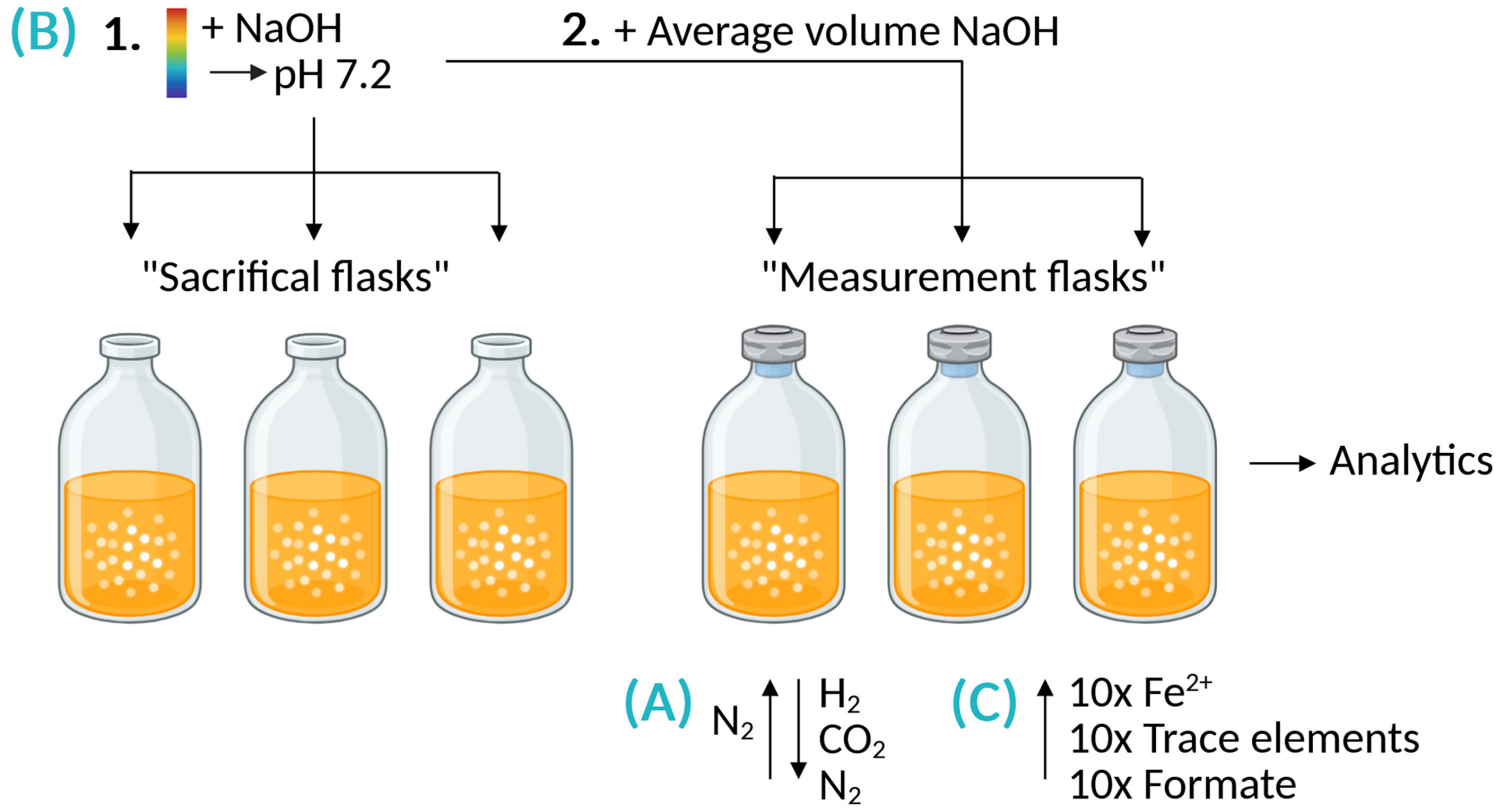
Schematic overview of inhibitor mitigation strategies. **(A)** N_2_ sparging to lower H_2_ partial pressure. **(B)** Two-step pH control strategy using sacrificial flasks to determine the required NaOH addition and subsequent addition of the average volume to measurement flasks used for analytics. **(C)** 10-fold supplementation of Fe^2+^, SL-10 trace elements, and formate. For details refer to 2.2.2.1–2.2.2.4.

To identify factors that inhibit H_2_ production and complete carbon source degradation, cultivation was prolonged to 11 days in targeted experiments to allow corrective measures to manifest. In these experiments, metabolic progress was assessed primarily from soluble substrate consumption and product formation in the liquid phase and not H_2_ production. Figure 2 schematically summarizes the workflows.

##### 2.2.2.1 Effect of H_2_ partial pressure control by N_2_ sparging

To evaluate the impact of headspace H_2_ accumulation, serum flask headspaces were sparged with 120 mL N_2_ gas on days 2 through 10 (Figure 2A). Sparging was performed by inserting two sterile needles through the septum (inlet connected to N_2_ through a sterile filter; outlet as a vent) to maintain anoxic conditions while displacing headspace gases. After sparging, headspace pressure was equilibrated to atmospheric pressure.

##### 2.2.2.2 Small-scale pH control

A two-step procedure was employed in order to evaluate the effect of the acidifying pH. This procedure enabled replicate comparability, minimized oxygen ingress, and allowed day-wise corrections without repeatedly opening of serum flasks (Figure 2B):

1. On each cultivation day from day 2 through day 10, three “sacrificial flasks” per condition were opened. The pH was measured with a calibrated pH electrode, and each sacrificial flask was adjusted to pH 7.2 ± 0.1 by dropwise addition of 1 M NaOH solution while gently mixing. Sacrificial flasks were not used for analytical measurements thereafter.
2. The mean NaOH volume, as determined from the sacrificial flasks on the respective day, was subsequently added to the closed “measurement flasks” of the same condition using sterile filters and syringes, without opening the bottles. The measurement flasks were designated for the purposes of sampling and analytics. The same average NaOH volume was also added to each set of sacrificial flasks prepared for the following daily pH assessment, in order to ensure that sacrificial and measurement flasks were kept on identical pH control schedules. Cumulative NaOH additions per bottle were recorded and included in following calculations. The total added volume was consistently below 3% v/v of the working volume in all experiments, thereby exerting a negligible effect on the composition of the medium and its salinity. Equal pH of exemplary unused sacrificial flasks was validated after completed cultivation.

##### 2.2.2.3 Combined N_2_ sparging and pH control

The combined effect of lowering H_2_ partial pressure and maintaining pH at 7.2 ± 0.1 was assessed by applying both interventions in parallel from day 2 through day 10. On each applicable day, N_2_ sparging (Section 2.2.2.1) was performed first, followed immediately by pH correction (Section 2.2.2.2).

##### 2.2.2.4 Trace element, iron, and formate supplementation

In a separate set of cultures, the concentrations of SL-10 trace elements, ferrous iron (as FeCl_2_·4H_2_O), and formate were increased 10-fold relative to the basal medium (Figure 2C). Cultivation was terminated after no further increase in headspace H_2_ was observed between day 4 and day 8.

#### 2.2.3 Optimization of controlled pH

To identify the optimal pH for H_2_ production under controlled conditions, the initial pH was set to 6.0–9.0 in 0.5 unit increments. During cultivation, the pH was maintained at the respective initial setpoint using the daily control procedure outlined in Section 2.2.2.2. In a subsequent experiment, increased carbon concentrations of 166.5 and 333.1 mmol L^−1^ were supplied as lactose at a controlled pH of 7.2 ± 0.1. In these experiments, cultivation was prolonged to 11 days and pH control was conducted on days 2 through 10.

### 2.3 Analytical methods

H_2_ in the headspace was quantified by gas chromatography (GC2010, Shimadzu, Austria) equipped with a thermal conductivity detector, following an established method (Wagner et al., 2019). Gas samples of 1 mL were withdrawn using syringes and measured directly at days 4 and 8, and optionally at days 2 and 11. H_2_ production was calculated using the ideal gas law while correcting for measured overpressure with a pressure gauge (G 1113, Senseca Germany GmbH). The calculation also accounted for headspace volume (including sampling and NaOH volumes), temperature, and atmospheric pressure to estimate the moles of H_2_ (Wagner et al., 2019; Wunderer et al., 2022).

Concentrations of carbon sources and fermentation products, including formate, lactate, acetate, and ethanol, were quantified at days 0, 2, 4, 8, and optionally 11 using an UltiMate 3000 HPLC system (Thermo Fisher Scientific, Austria) with a refractive index detector (RefractoMax521, Thermo Fisher Scientific, Austria). Separation was performed on a Rezex ROA Organic Acid H^+^ column (8%), 300 × 7.8 mm (Phenomenex, Germany). Samples were centrifuged for 10 min at 16100 × g and filtered through 0.22 μm syringe filters before injection. The mobile phase was 5 mmol L^−1^ sulphuric acid and separation was performed at 70 °C and 0.5 mL min^−1^. Calibration used external standards prepared from the carbon sources present in the cultivation medium and a mixed volatile acid standard solution (Sigma Aldrich, Germany).

The pH was measured prior to and after cultivation, and if applicable in sacrificial flasks during the cultivation, using a SevenExcellence pH meter (Mettler Toledo, Austria) with temperature compensated electrodes. pH electrodes were calibrated daily using a three-point calibration procedure.

Biomass formation was estimated *via* total protein quantification using a modified Bradford assay (Quick Start Bradford Protein Assay, Bio-Rad Laboratories, Germany). After cultivation, the entire culture broths were centrifuged in two steps for 15 min at 4637 × g (Rotanta 460 R, Andreas Hettich GmbH, Germany). Supernatants were discarded. Cell pellets were resuspended and lysed in MN Bead Tubes Type C (Macherey-Nagel, Germany) with 0.8 mL lysis buffer containing 25 mmol L^−1^ Tris HCl, 150 mmol L^−1^ NaCl, 1 mmol L^−1^ EDTA, and 5% glycerol at pH 7.4. Bead milling was performed for 5 min at 30 Hz using a MM 400 bead mill (Retsch GmbH, Germany). Lysates were mixed 1:1 with Quick Start Bradford 1 × Dye Reagent, incubated for 5 min at room temperature, and absorbance was measured at 595 nm using a Jenway 7,315 spectrophotometer (Fisher Scientific GmbH, Germany). Protein concentrations were calculated from a bovine serum albumin standard curve spanning 1.25–10 μg mL^−1^.

### 2.4 RNA extraction and real time quantitative PCR (RT-qPCR)

RT-qPCR was utilized to assess the relative expression of genes associated with the FHL pathway. The cDNA of *T. mirandus* was analyzed after a 4- and 8-day cultivation period without and with pH control at a setpoint of 7.2. This analysis was used to monitor the expression of genes coding for the competing pathway enzymes L-lactate dehydrogenase (*ldh*) and formate C-acetyltransferase/ pyruvate formate lyase (*pflB*). The hydrogenase formation protein HypD (*hypD*) was chosen as central FHL maturation marker (Nutschan et al., 2019; Watanabe et al., 2012). The total RNA was extracted from three whole replicate serum flasks by means of centrifugation for 2 min at 4637x g (Rotanta 460 R, Andreas Hettich GmbH, Germany). Samples were diluted in 1:1 Monarch DNA/RNA Protection Reagent (New England Biolabs GmbH, Germany) and frozen at −80 °C until further processing. The total RNA was extracted in accordance with the TRIzol™ Reagent protocol (Thermo Fisher Scientific, Austria). The obtained RNA pellet was collected in nuclease-free reaction tubes (Biozym, Germany) and dissolved in UltraPure™ DNAse/RNAse-free distilled water (Thermo Fisher Scientific, Austria). The final RNA concentration was measured using the NanoDrop™ 2000c spectrophotometer (Thermo Fisher Scientific, Austria). In order to synthesize cDNA, 1 μg of RNA from each sample was utilized, employing the iScript kit (Bio-Rad Laboratories, Austria) and Mastercycler^®^ nexus X2 (Eppendorf, Germany). The resulting cDNA was purified using the HiYield^®^ PCR Clean-up/Gel Extraction Kit (Süd-Laborbedarf GmbH, Germany). In the subsequent RT-qPCR reactions, a volume of 4 μL purified cDNA template, 250 nM of each primer and 10 μL of SsoAdvanced Universal SYBR Green Supermix (Bio-Rad Laboratories, Austria) were utilized in a duplicate reaction volume of 20 μL. No-reverse-transcriptase and no-template controls were included for each primer set to check for gDNA and nucleic acid contamination respectively. Amplification was performed in hard-shell PCR plates (Bio-Rad Laboratories, Austria) using a CFX96™ Real-Time System and C1000 Touch Thermal Cycler (Bio-Rad Laboratories, Austria). The cycling parameters comprised an initial 95 °C DNA polymerase activation step for 30 s, followed by 40 cycles of 95 °C for 15 s (denaturation), 60 °C for 30 s (annealing and extension) and a melt curve analysis to check for nonspecific amplification products (65–95 °C in 0.5 °C steps at 5 sec/step). Primer pairs were designed using Primer-BLAST by the National Library of Medicine for the investigated genes and are listed in Supplementary Table S5. Samples of the amplified sequences were subjected to sequencing using the forward primer and Mix2Seq kit (Eurofins, Austria), in order to confirm correct PCR product generation. Normalized expression of genes was calculated relative to the established housekeeping gene *gyrA* [Williams and Ghanem, 2022; DNA gyrase subunit A; fold change (FC) of C_q_ from no pH control to pH control at 7.2: 0.97 ± 0.08, *n* = 120].

### 2.5 Statistical analysis

Statistical analyses were conducted in GraphPad Prism 11 (GraphPad Software, USA). Unless stated otherwise, results are presented as mean ± standard deviation for *n* = 3 biological replicates. Depending on the comparison, two-tailed t-tests or one-way ANOVA were applied with Dunnett's multiple comparison test for comparisons to a reference and Tukey's test for pairwise group comparisons. A value of 0.05 was set for α. FC was calculated as the ratio of the mean in the test condition to the mean in the reference condition. The respective error (U) was calculated according to the Gaussian law of propagation of uncertainty.

## 3 Results

### 3.1 Fermentation characteristics

Based on previous investigations with the selected organism, *T. mirandus* was cultivated in a defined medium containing lactose as the carbon source and yeast extract as the nitrogen source for 8 days to characterize H_2_ production, growth (approximated by total protein), pH development, lactose degradation, and metabolite formation, and to establish a baseline for optimization. Protein and metabolite concentrations were corrected by subtracting the respective contents of the uninoculated medium.

**Figure 3:**
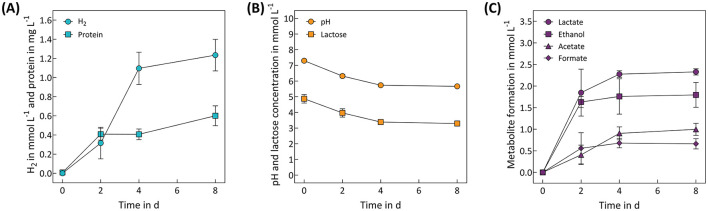
Fermentation parameters of a *T. mirandus* batch cultivation for 8 days in lactose–yeast extract medium. **(A)** Cumulative H_2_ production and protein concentration over time (mean ± SD). **(B)** Time courses of pH and lactose concentration (mean ± SD). **(C)** Endpoint metabolite concentrations (lactate, ethanol, acetate, and formate; mean ± SD).

The largest changes in all measured parameters occurred within the first 4 days, after which most trajectories plateaued or changed only slightly (Figure 3). After 8 days, cumulative H_2_ production reached 1.2 ± 0.2 mmol L^−1^, corresponding to 136.7 ± 9.2 mmol mol hexose equivalent^−1^, and protein reached 0.60 ± 0.10 g L^−1^. The maximum H_2_ production rate (VHPR) was achieved between days 2 and 4 (0.30 ± 0.05 mmol H_2_ L^−1^ d^−1^) and the maximum substrate consumption rate (VSCR) between days 0 and 2 (0.46 ± 0.28 mmol lactose L^−1^ d^−1^). The pH decreased from 7.3 ± 0.1 to 5.7 ± 0.1. Of the initially supplied lactose (4.9 ± 0.3 mmol L^−1^), 32.1 ± 7.1% was consumed, equivalent to 1.6 ± 0.4 mmol L^−1^ lactose degraded. The primary fermentation products were lactate (2.3 ± 0.1 mmol L^−1^, 0.24 ± 0.01 mol mol hexose equivalent^−1^), ethanol (1.8 ± 0.3 mmol L^−1^, 0.18 ± 0.02 mol mol hexose equivalent^−1^), acetate (1.0 ± 0.1 mmol L^−1^, 0.10 ± 0.02 mol mol hexose equivalent^−1^), and formate (0.7 ± 0.1 mmol L^−1^, 0.07 ± 0.02 mol mol hexose equivalent^−1^) in decreasing order of concentration. CO_2_ was detected by gas chromatography but was not quantified due to its pH dependent solubility.

### 3.2 Substrate utilization

**Figure 4:**
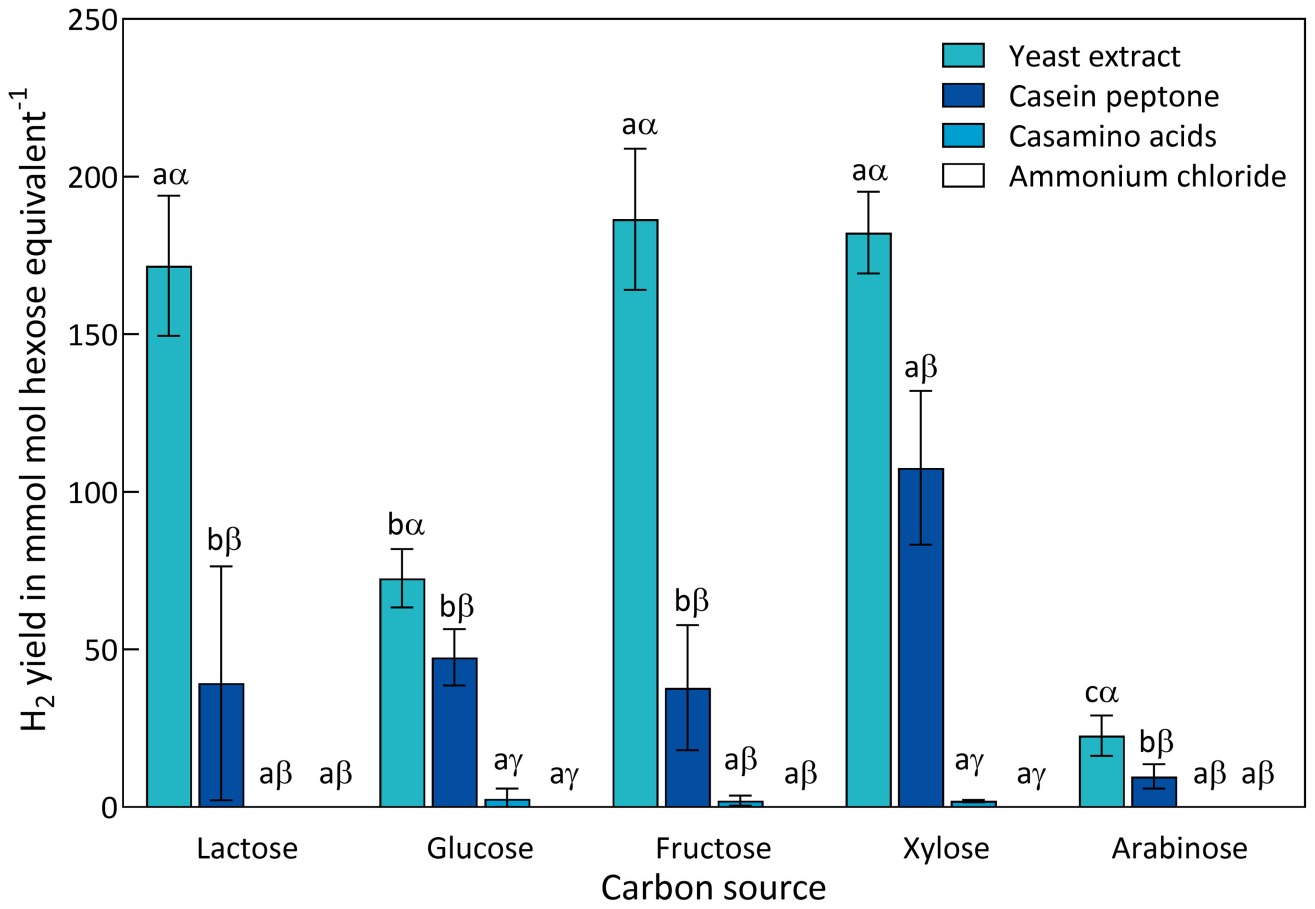
Average H_2_ yield (mean ± SD) for all combinations of carbon sources (lactose, glucose, fructose, xylose, and arabinose) and nitrogen sources (yeast extract, casein peptone, casamino acids, and ammonium chloride). Significant differences (*p* ≤ 0.05) are indicated by different characters. Lower case characters indicate comparison of carbon sources with equal nitrogen source and Greek characters comparison of nitrogen sources with equal carbon sources.

After establishing a reproducible reference cultivation process using lactose, the utilization of other carbon and nitrogen sources was assessed in combination. These were selected based on frequent use according to literature (Ergal et al., 2018; Ferchichi et al., 2005; Hamilton et al., 2018; Łukajtis et al., 2018) and their prevalence in food and agricultural residues which are commonly valorized for a more economically feasible and sustainable H_2_ production (Ferchichi et al., 2005; Łukajtis et al., 2018; Soares et al., 2020). Figure 4 summarizes the average H_2_ yield for all combinations at equal initial carbon concentrations of 66.6 mmol L^−1^ which was selected based on the media composition in the preceding study with *T. mirandus* by Mutschlechner et al. (2021). A C/N ratio of 20 and a respective nitrogen concentration of 2.9 mmol L^−1^ was chosen as an average value of the reported optimal range of C/N from 6.7 to 200 (Anzola-Rojas et al., 2015; Argun et al., 2008; Hamilton et al., 2018; Lin, 2004; Liu and Shen, 2004; Morimoto, 2004).

Across all nitrogen sources, the complex nitrogen source yeast extract significantly supported the highest H_2_ yields (*p* ≤ 0.05), with a maximum of 186.5 ± 22.4 mmol mol hexose equivalent^−1^ for fructose. Using glucose as an example, H_2_ yield decreased in the order: yeast extract > casein peptone > casamino acids ≈ ammonium chloride, with no measurable H_2_ production when ammonium chloride was the sole nitrogen source. Differences among nitrogen sources were significant in each case (*p* ≤ 0.01), except between casamino acids and ammonium chloride (*p* > 0.05). A similar pattern was observed for the other carbon sources, except that casein peptone and casamino acids/ammonium chloride did not differ significantly for lactose, fructose, and arabinose.

With yeast extract, the most efficient carbon sources for H_2_ production ranked as fructose ≈ xylose ≈ lactose > glucose > arabinose. Yields for fructose, xylose, and lactose did not differ significantly (*p* > 0.05) but each exceeded those for glucose and arabinose significantly (*p* ≤ 0.0001). Also, the VHPR with fructose and xylose during the first four cultivation days was significantly higher than with any other carbon source (*p* ≤ 0.05; Supplementary Table S1). With casein peptone and casamino acids, the rank order for H_2_ yield differed, and glucose was among the most efficient. Arabinose led to the lowest H_2_ yields for all nitrogen sources.

None of the carbon sources was completely degraded under these conditions (Supplementary Figure S1). Nevertheless, partial consumption of each carbon source occurred with every nitrogen source, even when H_2_ production was not detected. Fructose and xylose exhibited a higher rate of degradation and an earlier onset of degradation in comparison to the other carbon sources when combined with yeast extract (Supplementary Table S2). The maximum VSCR for lactose was only reached between cultivation days 2 and 4 and glucose degradation had already mainly ceased by day 2.

No clear preference for hexoses vs. pentoses emerged, since xylose (pentose) and fructose (hexose) outperformed arabinose (pentose) and glucose (hexose) in terms of H_2_ production, VHPR and VSCR.

### 3.3 Optimization of carbon to nitrogen ratio and carbon concentration

**Figure 5:**
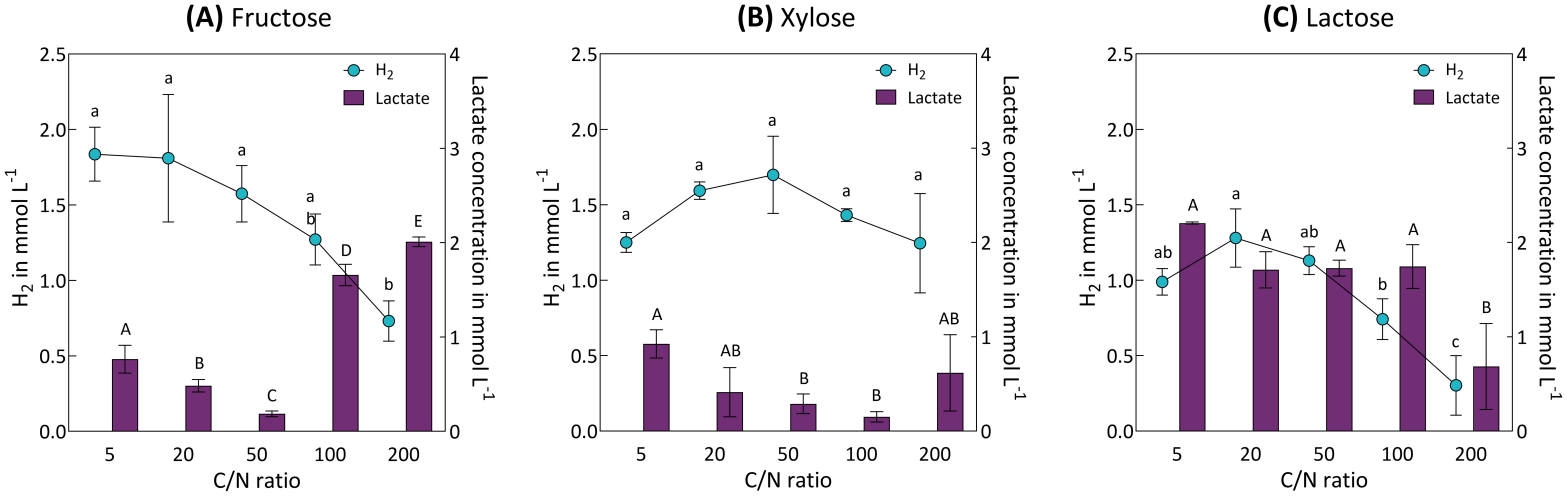
Effects of C/N ratio on H_2_ and lactate production (mean ± SD) during a *T. mirandus* fermentation over 8 days with yeast extract as nitrogen source, three different carbon sources and a carbon concentration of 66.6 mmol C L^−1^. Significant differences (*p* ≤ 0.05) are indicated by different characters. Lower case characters indicate comparison of H_2_ production and upper case characters comparison of lactate production. **(A)** Fructose. **(B)** Xylose. **(C)** Lactose.

Based on the screening described above, fructose, xylose, and lactose with yeast extract were selected for optimization of H_2_ production. The effect of the C/N ratio (5, 20, 50, 100, and 200) at three initial carbon concentrations (66.6, 166.5, and 333.1 mmol L^−1^) on H_2_ production was examined. The H_2_ yield per hexose equivalent is shown in Supplementary Figure S2 and the volumetric H_2_ production and lactate concentration for 66.6 mmol C L^−1^ is displayed exemplary in Figure 5. Furthermore, protein formation, carbon source utilization, and the concentrations of acetate and ethanol were quantified and are displayed for each carbon concentration in Supplementary Figures S3, S4 and S5. The VHPR and VSCR values are displayed exemplarily for the carbon concentration 66.6 mmol C L^−1^ in Supplementary Tables S3, S4.

In general, H_2_ yield decreased with increasing initial carbon concentrations. The C/N ratio that maximized H_2_ production depended on the carbon source. For fructose at 66.6 mmol L^−1^, lower C/N ratios (5–50) supported the highest H_2_ production, whereas higher C/N ratios (50–200) were more favorable at 166.5 and 333.1 mmol C L^−1^. Xylose showed similar tendencies, with the highest H_2_ production at C/N ratios between 20 and 100 for 66.6 mmol C L^−1^. By contrast, with lactose at 66.6 and 333.1 mmol C L^−1^, lower C/N ratios (5–50) were beneficial. At 333.1 mmol C L^−1^ lactose, the extreme ratios 5 and 200 supported higher H_2_ production than the intermediate ratios.

At 66.6 mmol C L^−1^, growth (i.e., protein concentration) corresponded to H_2_ production across C/N ratios for all three carbon sources, except for low C/N ratios with fructose for which H_2_ increased but protein concentration decreased. Protein concentrations were lower with xylose than with fructose or lactose, despite similar H_2_ production was found. None of the carbon sources was fully consumed under any condition, and the extent of substrate depletion mirrored H_2_ production except at low C/N with fructose. Also the VHPR and VSCR values were distributed equally to the volumetric H_2_ production. Nevertheless, the degradation of xylose was found to occur at its maximum rate during the first two cultivation days. In contrast, the consumption of fructose was found to be predominant during days 2–4, while lactose underwent degradation primarily during days 2–8. Metabolite patterns showed inverse relationships between lactate and H_2_ for fructose and xylose, but not with lactose. Acetate and ethanol formation generally increased in parallel with H_2_ concentrations across all carbon sources. Comparable trends in growth, substrate consumption, and by-product formation were observed at the higher carbon concentrations.

### 3.4 Inhibiting factors

**Figure 6:**
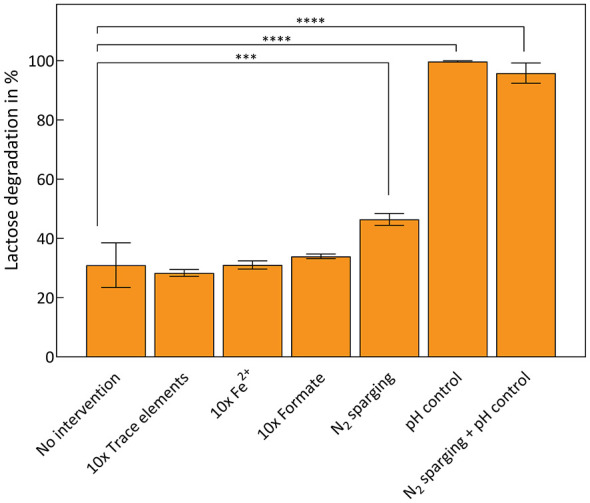
Potential inhibitory factors for lactose degradation (mean ± SD) by *T.mirandus*: 10-fold increase of trace element concentrations, iron and formate; daily N_2_ headspace sparging; daily pH adjustment to 7.2; and the combined N_2_ sparging plus pH control. Significance differences to controls are indicated as follows: ***, *p* ≤ 0.001; ****, *p* ≤ 0.0001.

Guided by the incomplete lactose degradation in baseline experiments, potential limiting factors for complete conversion were tested. Figure 6 shows lactose consumption under different interventions designed to alleviate putative constraints. Experiments were stopped after no further lactose degradation could be detected. Increasing the initial concentrations of trace elements, Fe^2+^, and formate by 10-fold did not change lactose degradation. Formate decreased by 2.4 ± 2.8 % from an initial concentration of 9.0 ± 0.1 mmol L^−1^. In contrast, daily sparging of the headspace with N_2_, daily pH adjustment, and the combination of both, resulted in a significant increase of lactose utilization and in the two latter cases to approximately complete conversion of lactose (for pH: 99.7 ± 0.2 %; for N_2_ + pH: 95.9 ± 3.4 %).

### 3.5 Cultivation with pH control

**Figure 7:**
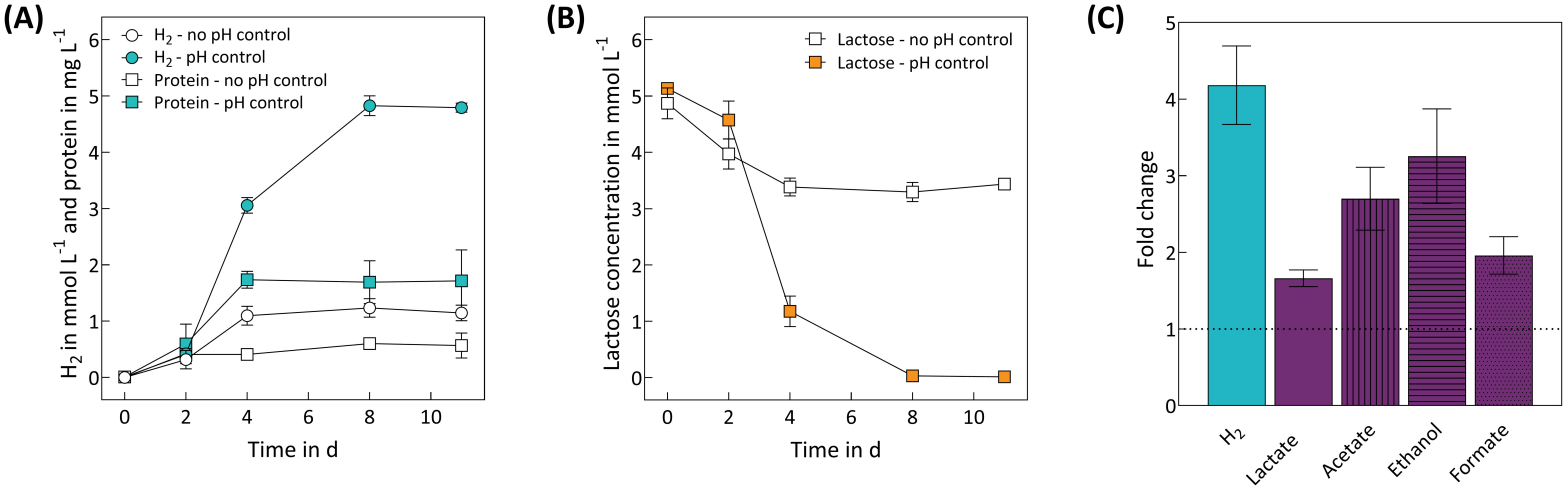
Effect of pH control at target pH 7.2 on *T. mirandus* fermentation over 11 days compared to no pH control (initial pH 7.2). **(A)** Cumulative H_2_ production and protein concentration (mean ± SD). **(B)** Lactose concentration (mean ± SD). **(C)** Fold change pH control vs. no control of H_2_ production and metabolite formation (lactate, ethanol, acetate, and formate) with values >1 as an increase, =1 for no change and <1 as an decrease (FC ± U).

**Figure 8:**
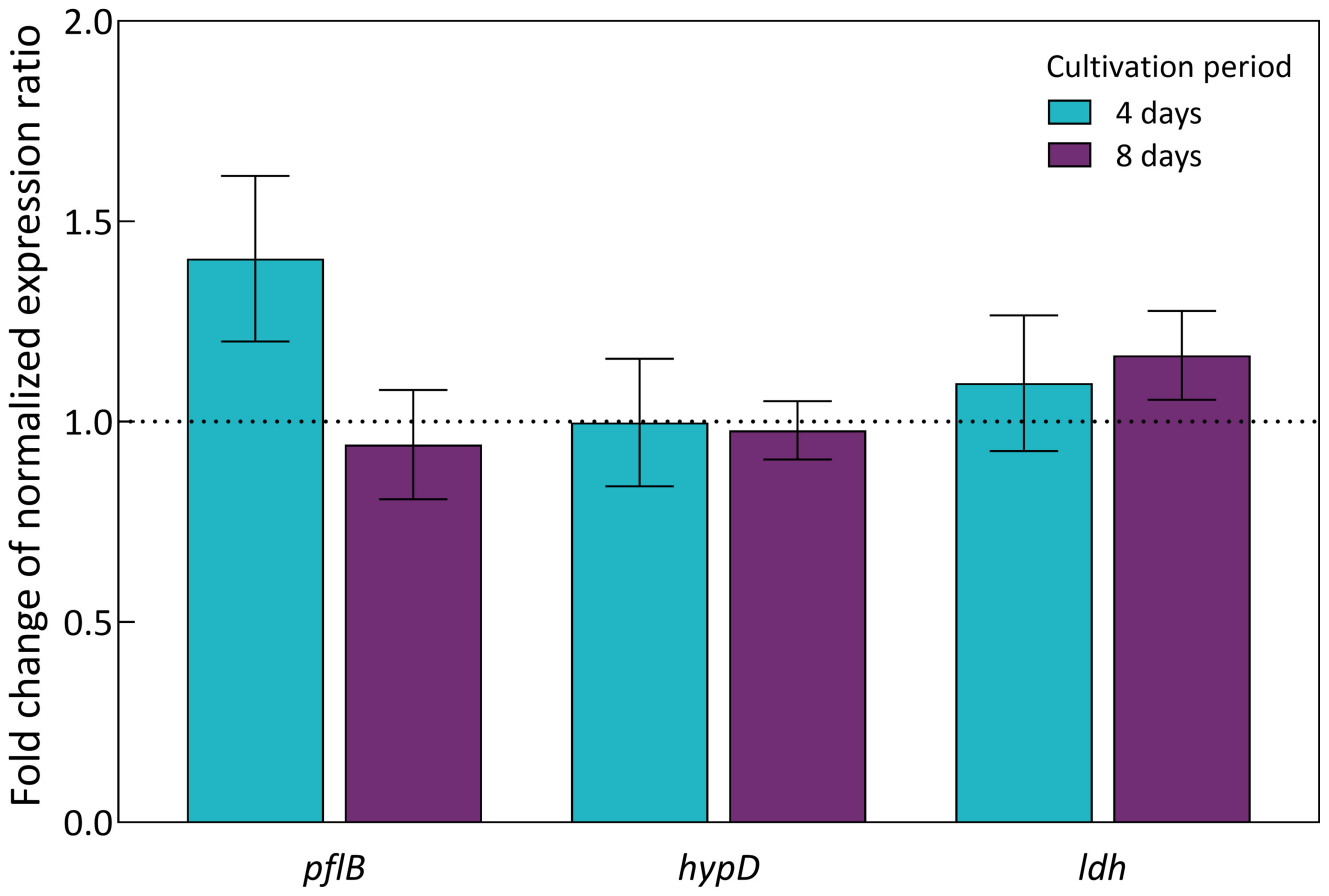
Effect of pH control at target pH 7.2 on FHL pathway gene expression in *T. mirandus* after a cultivation period of 4 and 8 days. Fold change pH control vs. no control of the normalized expression ratio of the genes *pflB, hypD* and *ldh* with *gyrA* as a reference gene and values >1 as an increase, =1 for no change and <1 as an decrease (FC ± U).

Motivated by the complete lactose degradation achieved *via* daily pH adjustment to 7.2, the effect of pH control was evaluated in detail. Figure 7 compares 11-day batch fermentations with and without daily pH control. After a lag phase until day 2, the highest VHPR and VSCR with pH control occurred between days 2 and 4 (0.83 ± 0.04 mmol H_2_ L^−1^ d^−1^; 1.70 ± 0.12 mmol lactose L^−1^ d^−1^), followed by a gradual approach to a plateau by day 8. Final H_2_ reached 4.79 ± 0.08 mmol L^−1^, equivalent to 466.6 ± 10.2 mmol mol hexose equivalent^−1^ after 11 days, a 4.2-fold increase over the no-control condition (*p* ≤ 0.0001; 314% increase). Protein plateaued by day 4 at 1.7 ± 0.55 g L^−1^ (3.0-fold increase vs. no pH control, *p* ≤ 0.05). Lactose consumption reached 99.4 ± 0.2% by day 8. All measured fermentation products increased significantly with pH control (*p* ≤ 0.001; FC >1), although to a lesser extent than H_2_ concentration. Figure 8 illustrates the impact of pH control on the FHL pathway gene expression as FC compared to no pH control conditions, while Supplementary Table S6 provides the normalized expression ratios. A significant upregulation of *pflB* (*p* ≤ 0.001; FC 1.4 ± 0.2) was observed after 4 days of cultivation with pH control. Furthermore, *ldh* was significantly upregulated in conjunction with pH control after an 8 days cultivation period (*p* ≤ 0.01; FC 1.2 ± 0.1). No change in normalized gene expression of *hypD* was found during the whole cultivation.

**Figure 9:**
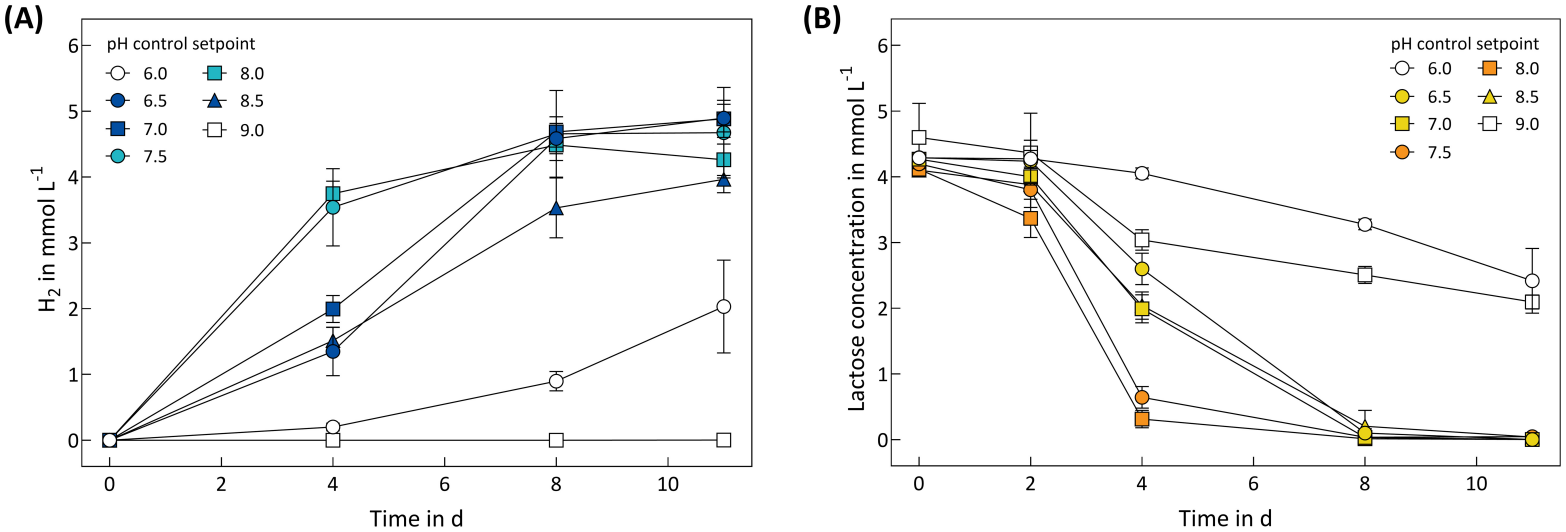
Batch fermentations with pH controlled at setpoints from 6.0 to 9.0 over 11 days. **(A)** Cumulative H_2_ production (mean ± SD). **(B)** Lactose concentration over time (mean ± SD).

Next, pH was controlled at fixed setpoints (target pH) from 5.0 to 9.0 in 0.5 pH unit increments. As shown in Figure 9, total H_2_ and complete lactose consumption after 11 days were similar for pH 6.5–8.5 samples (H_2_: *p* > 0.05; lactose: *p* > 0.05), but both were significantly lower at pH 6.0 (H_2_: *p* ≤ 0.01; lactose: *p* ≤ 0.0001) and pH 9.0 (H_2_: *p* ≤ 0.0001; lactose: *p* ≤ 0.0001). The VHPR between days 0 and 4 was significantly higher at pH 7.5 and 8.0 than at other setpoints (*p* ≤ 0.001), and only at these pH values the maximum VHPR was reached in the first 4 days (Supplementary Table S7). The VSCR was likewise significantly higher at pH 7.5 and 8.0 between days 2 and 4 (*p* ≤ 0.01, Supplementary Table S8). Supplementary Figure S7 shows no or only minor significant differences in end-point metabolite distributions and protein concentrations in the pH range between 6.5 and 8.5, with significant deviations only at pH 6.0 and 9.0. It was observed that among the metabolites, only formate concentrations were increased within the range of 7.5–8.5.

**Figure 10:**
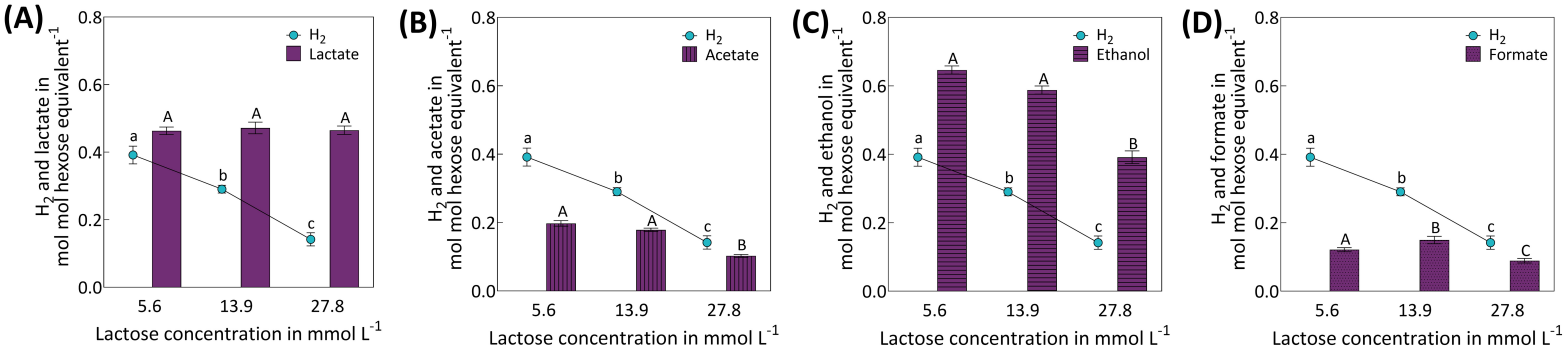
Fermentation product yields per hexose equivalent after 11 days with pH control at 7.2 and initial lactose concentration of 5.6, 13.9, and 27.8 mmol L^−1^. Significant differences (*p* ≤ 0.05) are indicated by different characters. Lower case characters indicate comparison of H_2_ production and upper case letter comparison of metabolite production. **(A)** H_2_ and lactate yield (mean ± SD). **(B)** H_2_ and acetate yield (mean ± SD). **(C)** H_2_ and ethanol yield (mean ± SD). **(D)** H_2_ and formate yield (mean ± SD).

Finally, pH controlled runs at setpoint 7.2 were repeated with higher initial lactose concentrations of 5.6, 13.9, and 27.8 mmol L^−1^ (i.e. 66.6, 166.5, and 333.1 mmol C L^−1^). Results are summarized in Table 1 and Supplementary Tables S9 and S10. The highest final H_2_ concentrations were obtained at 13.9 and 27.8 mmol L^−1^ lactose (*p* ≤ 0.0001), while the highest H_2_ yield occurred at 5.6 mmol lactose L^−1^ (*p* ≤ 0.01). Lactose consumption reached at least 94.8 ± 0.5% at all concentrations after 11 days. The maximum VHPR increased from 0.64 ± 0.03 mmol H_2_ L^−1^ d^−1^ between days 0 and 4 at 5.6 mmol lactose L^−1^ to 1.58 ± 0.06 mmol H_2_ L^−1^ d^−1^ between days 4 and 8 at 13.9 mmol lactose L^−1^ (*p* ≤ 0.0001, 147% increase). As shown in Figure 10, lactate concentrations were equally produced at every lactose concentration (*p* > 0.05) but dominated as a fermentation product only at 27.8 mmol lactose L^−1^, whereas ethanol dominated at 5.6 and 13.9 mmol lactose L^−1^. Ethanol and acetate yield increased significantly at 5.6 and 13.9 mmol lactose L^−1^ compared to 27.8 mmol lactose L^−1^ (*p* ≤ 0.0001). The highest formate concentration was found at 13.9 mmol lactose L^−1^.

**Table 1:** Fermentation performance (mean ± SD) of a 11-day *T. mirandus* cultivation with pH control at setpoint 7.2 at different initial lactose concentrations (5.6, 13.9, and 27.8 mmol L^−1^), compared to no pH control at 5.6 mmol L^−1^ (initial pH 7.2).

Lactose [mmol L^−1^]	pH control	H_2_ [mmol L^−1^] (end-point)	H_2_ yield [mmol mol hexose equivalent^−1^] (end-point)	VHPR_max_ [mmol L^−1^ d^−1^] (timeframe in d)	Lactose degradation [%] (end-point)	Protein [mg L^−1^] (end-point)
5.6	No	1.22 ± 0.17	143.47 ± 17.95	0.31 ± 0.65 (0–4 d)	29.5 ± 7.9	0.60 ± 0.10
5.6	Yes	4.24 ± 0.15	391.57 ± 26.14	0.64 ± 0.03 (0–4 d)	99.7 ± 0.1	1.80 ± 0.06
13.9	Yes	8.05 ± 0.22	290.48 ± 11.48	1.58 ± 0.06 (4–8 d)	99.4 ± 0.2	8.70 ± 1.10
27.8	Yes	7.50 ± 0.59	141.77 ± 19.48	1.40 ± 0.09 (4–8 d)	94.8 ± 0.5	14.16 ± 1.52

In brackets it is indicated if the parameters are end-point values or applicable for a certain timeframe.

## 4 Discussion

### 4.1 Fermentation characteristics

Batch cultivations with the newly discovered thermophilic anaerobe *T. mirandus* were conducted to characterize its H_2_ production, identify key factors for optimization, and uncover principles that could inform H_2_ yield improvements in diverse DF processes. In the liquid and gas phase of batch cultures, the detected metabolites comprised H_2_, CO_2_, lactate, ethanol, acetate, and formate. This pattern, together with a gene cluster encoding a structural [NiFe] hydrogenase and its assembly and maturation proteins (*hypABCDEF*), supports operation of a FHL pathway in *T. mirandus*, consistent with the genome-based analysis reported by Mutschlechner et al. (2021) and the pathway schematized in Figure 1. All indicated genes were identified from the genome of *T. mirandus* (NCBI asssembly ASM1386805v1, National Library of Medicine).

During a baseline cultivation process, the theoretical H_2_ yield of 2 mol H_2_ per mol hexose equivalent for microorganisms utilizing the FHL pathway (Cao et al., 2022) was not achieved under the set cultivation conditions showing the need for further process optimization. Potential causes for the low yield included incomplete lactose utilization that limited biomass formation, or diversion of pyruvate to lactate. Candidate process variables affecting these outcomes included substrate type, C/N ratio, pH, temperature, H_2_ partial pressure, metal ions and formate concentration (Chen et al., 2021; Sawers, 2005; Soares et al., 2020; Zhang et al., 2020). As optimal growth and H_2_ production at 55 °C were demonstrated previously for this strain (Mutschlechner et al., 2021), subsequent work focused on the remaining factors.

### 4.2 Substrate utilization

Consistent with Mutschlechner et al. (2021), the strain utilized lactose and glucose; however, based on the present data, fructose and xylose can be added to the substrate portfolio of *T. mirandus*. Substrate preference is known to be strain dependent. For example, strains of *Clostridium butyricum* produced larger H_2_ volumes from sucrose, glucose and fructose than from lactose, with the highest from potato starch and molasses (Wang et al., 2008). In mixed cultures, hydrolysis of di- and trisaccharides prior to fermentation can negatively influence H_2_ yield (Quéméneur et al., 2011). Conversely, disaccharides such as sucrose and lactose yielded higher H_2_ concentrations than monosaccharides such as glucose and fructose in some pure cultures, including mesophilic *Clostridium saccharoperbutylacetonicum* (Ferchichi et al., 2005) and thermophilic *Thermoanaerobacterium thermosaccharolyticum* (Litti et al., 2022). These reports underscore that substrate effects are organism and process specific.

Among nitrogen sources, casein peptone and yeast extract supported growth of *T. mirandus*. Moreover, yeast extract resulted in significantly higher H_2_ production than casein peptone, likely due to its complex composition that supplies essential amino acids, vitamins, nucleosides, peptides, and minerals (Tao et al., 2023). Growth requirements for specific vitamins have been shown for several anaerobes, for example in *Clostridium perfringens* supplied with adenine, calcium pantothenate, pyridoxine, and biotin (Fuchs and Bonde, 1957). Casein peptone and casamino acids derive from casein, but enzymatic digestion vs. acid hydrolysis generates peptide rich vs. amino acid rich compositions with different availabilities to anaerobes (Attwood et al., 1998; Takahashi et al., 2000; Wagner et al., 2012). Negative effects of inorganic nitrogen sources on H_2_ production or VHPR have also been reported for DF (Alvarado-Cuevas et al., 2013; Ferchichi et al., 2005; Hamilton et al., 2018; Rorke et al., 2022), presumably due to additional energetic costs for *de novo* amino acid synthesis (Ferchichi et al., 2005; Hamilton et al., 2018). On this basis, yeast extract was supplied as the nitrogen source for subsequent experiments. The carbon contribution from nitrogen supplements was not considered in calculations because they were equal for each carbon source when being combined with a nitrogen source.

Degradation of the carbon source was measured in some substrate combinations associated with no or very low H_2_ production, for example every combination with arabinose, casamino acids and ammonium chloride. This suggests that *T. mirandus* can metabolize these substrates, but that specific carbon and nitrogen source combinations do not provide conditions conducive to H_2_ production.

Overall, these results illustrate strain specificity and show application potential in waste valorization. Simple sugars are valuable for mechanistic studies and strain screening but their use at scale is not economically viable (Ferchichi et al., 2005; Łukajtis et al., 2018; Soares et al., 2020). Industrial side streams, including lactose-rich cheese whey and lignocellulosic biomass hydrolysates have been successfully valorized for bio-H_2_ production (Alvarado-Cuevas et al., 2013; Cardeña et al., 2025; Kargi et al., 2012). The ability of *T. mirandus* to produce H_2_ from lactose, glucose, fructose and xylose, in combination with yeast extract or casein peptone, provides a basis for future application of complex substrate thus for utilizing, for example, cheese whey (lactose and casein peptone), food waste (fructose and glucose) and/or lignocellulosic hydrolysates (xylose and glucose). Supplementation with yeast extract or similarly rich nitrogen sources may be required to enable sufficient growth and H_2_ production, as also demonstrated for cheese whey fermentation by *E. coli* (Alvarado-Cuevas et al., 2013).

### 4.3 Optimization of carbon to nitrogen ratio and carbon concentration

Across carbon sources and concentrations, the tested C/N ratios yielded limited statistically significant differences, although consistent tendencies emerged for each carbon source and concentration. The tested ratios were selected based on reported optima for DF pure and mixed cultures, spanning approximately 6.7–200 (Anzola-Rojas et al., 2015; Argun et al., 2008; Hamilton et al., 2018; Lin, 2004; Liu and Shen, 2004; Morimoto, 2004). In cases where H_2_ production increased at the lowest or highest tested C/N ratio, additional testing below or above this common range could further improve H_2_ production. Examples include fructose at 66.6 and 333.1 mmol C L^−1^, xylose at 166.5 and 333.1 mmol C L^−1^ and lactose at 333.1 mmol C L^−1^. The identification of suitable C/N ratios is of particular relevance in the context of selecting waste substrates with suitable C/N ratios. In such scenarios, the limited influence of the C/N ratio on H_2_ production is advantageous. The co-fermentation of complementary waste or the addition of external nitrogen sources to achieve a target C/N ratio is a possibility, but it increases the complexity of the process (Alvarado-Cuevas et al., 2013; Reyna-Gómez et al., 2019; Zhang et al., 2020).

For fructose and xylose, increased H_2_ production coincided with increased acetate and ethanol formation and reduced lactate, suggesting shifts in pathway activity with C/N. Acetate synthesis is generally favorable for H_2_ production (Soares et al., 2020), and C/N driven increases in acetate have been positively correlated with H_2_ production (Zhang et al., 2020). In organisms that use PFL and FHL pathways, equimolar ethanol and acetate can be generated with a net gain of one ATP per pyruvate, which links increased ethanol to increased acetate and H_2_ (Hasona et al., 2004). Consistent with the pathways depicted in Figure 1, lactate formation competes with H_2_ production, so lower lactate is beneficial (Soares et al., 2020).

With lactose as the carbon source, H_2_ production depended on C/N, but the proportions of lactate, acetate and ethanol remained approximately scaled in parallel with H_2_. Genome analysis of *T. mirandus* with the tool BlastKOALA (Kanehisa et al., 2016) suggests different net ATP yields across sugars. For xylose, transport *via* a xylose transporter (*xylE*) does not directly hydrolyze ATP, and conversion through xylose isomerase (*xylA*) and xylulokinase (*xylB*) into the non-oxidative pentose phosphate pathway yields approximately 1.67 ATP per mol xylose. Fructose is presumably imported through a phosphotransferase system IIABC as fructose-1-phophate, consuming one phosphoenolpyruvate (PEP). After conversion to fructose-1,6-diphosphate by a 1-phosphofructokinase (*fruK*) it can be assumed to enter glycolysis, with a net yield of 2 ATP per mol fructose. Lactose may be transported as lactose-6-phosphate *via* a phosphotransferase IIABC transporter, consuming one PEP, then split into glucose and galactose-6-phosphate by a 6-phospho-β-glucosidase. The enzyme presumably has both phosphor-β-glucosidase and phosphor-β-galactosidase activity as described for *Lactococcus lactis* (Aleksandrzak-Piekarczyk et al., 2005), since no dedicated phosphor-β-galactosidase was identified in the genome. Separate metabolization of glucose and galactose, possibly *via* glycolysis and the tagatose-6-phosphate pathway due to presence of *lacABCD*, would yield approximately 4 ATP per mol lactose.

In theory, equal ATP yields per total carbon would be expected when carbon concentrations are normalized by carbon atom number, but only if sugars are consumed completely. Observed incomplete consumption, different ATP yields per mol substrate and potentially different uptake or conversion rates could bias metabolism toward acetate formation as a route to additional ATP *via* ACK, with this tendency decreasing from xylose to fructose to lactose. This may also explain elevated VSCR values and the accelerated onset of substrate degradation but at the same time lower growth on xylose compared with fructose and lactose. Similar constraints have been reported, for example anaerobic growth of *E. coli* relies on activity of PFL and ACK to gain ATP relative to glucose metabolism when processing xylose, compensating for phosphorylation, and in the case of *E. coli*, transport costs (Hasona et al., 2004). Under carbon limiting conditions with less favorable sugars, *Lactococcus lactis* shifts fermentation from lactate toward acetate and ethanol, reflecting an alleviation of PFL inhibition (due to lower levels of glyceraldehyde-3-phosphat and dihydroxyacetone phosphate) and reduced activation of LDH (by less frutcose-1,6-diphosphate and/or NADH; Collet et al., 2006; Melchiorsen et al., 2001).

By integrating genome analysis with the observed metabolite trends across C/N ratios, the pathways most likely responsible for H_2_ synthesis were elucidated. This not only improves process understanding and enables prediction of byproduct formation, but also provides a basis for process optimization and substrate selection.

Complete sugar degradation was not achieved for any sugar, concentration or C/N ratio in batch, which precludes robust comparison across carbon concentrations at this stage. This motivated an in depth investigation of potential inhibitory factors. A C/N ratio of 20 was used for subsequent work because it supported H_2_ production with lactose at 66.6 mmol C L^−1^ with yeast extract.

### 4.4 Inhibiting factors

No deficiencies in trace elements, Fe^2+^ or formate were identified that could explain the incomplete lactose degradation observed in basal experiments. N_2_ headspace sparging affected lactose degradation, but its impact was minor compared to pH control, which emerged as the decisive factor and was thus investigated further. A low pH can result in the collapse of pH homeostasis and incomplete depletion of substrates (Bowles and Ellefson, 1985; Täuber et al., 2021). Some measures may still contribute beneficially in combination with pH control, which was not evaluated here. For instance, metal ion concentrations in the basal medium were below reported optimal levels and far from reported inhibitory thresholds for mixed cultures and *E. coli*, respectively. In this study, Fe^2+^ was increased from 0.823 to 8.23 mg L^−1^. Reported optimum ranges include approximately 10–589.5 mg L^−1^ for mixed cultures (Wang and Wan, 2009) and >1.7 mg L^−1^ for *E. coli* (Kim et al., 2010), with inhibitory ranges reported between 8 and >50 mg L^−1^ depending on system and conditions (Chen et al., 2021). The same applies to formate which was shown to stimulate H_2_ evolution by serving as substrate and by inducing FHL gene expression (Pinske and Sawers, 2016; Rossmann et al., 1991; Sawers, 2005). In addition, higher H_2_ partial pressure (10%−30%) can exert a moderate inhibitory effect on H_2_ production, and a pronounced effect at concentrations exceeding 30% (Das et al., 2020). Strategies to remove H_2_ from the headspace, such as N_2_/CO_2_ sparging, stirring, or continuous gas stripping, can therefore enhance H_2_ production (Chou et al., 2008; Kim et al., 2023; Mizuno et al., 2000; Nemestóthy et al., 2020). In the present study, no H_2_ concentrations greater than 10% were measured, consistent with the minor effect of N_2_ sparging and indicating that thermodynamic inhibition was limited under these conditions compared to kinetic control via pH. Given the significant improvement of lactose degradation with pH control, N_2_ sparging may have a greater impact at higher lactose loadings that drive H_2_ above 10%.

### 4.5 Cultivation with pH control

Both initial pH (Khanal, 2004; Kumar and Das, 2000) and controlled pH (Dong et al., 2025; Li and Fang, 2007; Łukajtis et al., 2018) are recognized as critical parameters in DF. This was confirmed for *T. mirandus* by a 4.2-fold increase in volumetric H_2_ production and complete lactose degradation when pH was controlled at 7.2. In a study with *Clostridium sp*., H_2_ yield increased 1.3-fold with pH control at 6.0 for xylose and glucose during continuous fermentation (Taguchi et al., 1995). For *C. butyricum*, pH control at 5.5 increased H_2_ yield from 0.61 to 2.78 mol H_2_ mol sucrose^−1^, a 4.6-fold improvement (Chen et al., 2005), similar in magnitude to the improvement observed here. The obtained maximum H_2_ yield corresponds to 23% of the maximum FHL-pathway yield of 2 mol mol hexose equivalent^−1^ indicating carbon retention in by-products such as lactate, acteate, ethanol, and residual formate. DF could be coupled with a second stage (e.g., methanogenesis or further H_2_ conversion) to raise overall energy recovery beyond the direct H_2_ fraction observed (Ergal et al., 2022; Saidi et al., 2023; Zagrodnik and Duber, 2024).

Ethanol and, to a lesser extent, acetate increased in parallel with enhanced cell growth and H_2_ production under pH control, whereas lactate and formate increased by a lower factor relative to H_2_. One explanation involves enzyme pH optima. LDH optima have been reported near pH 6.0–6.5 in *Clostridium thermolacticum* (Collet et al., 2006) and near 5.5 in *Streptococcus bovis* (Asanuma and Hino, 2000). The [NiFe] hydrogenase Hyd 3 and the full FHL complex in *E. coli* are active at mildly acidic pH around 6.5, consistent with a role in relieving formate accumulation (Ergal et al., 2018; Trchounian et al., 2012; Yoshida et al., 2005). In contrast, reported PFL optima cluster around 7.5–8.5 in *E. coli* and *S. bovis* (Asanuma and Hino, 2000; Knappe et al., 1974). Lower intracellular pH would directly activate LDH and could reduce PFL activity, leading to accumulation of fructose-1,6-diphosphate and NADH and thereby further activating LDH (Collet et al., 2006). *pflB* upregulation with pH control on day 4 indicates strengthened capacity for PFL-mediated conversion of pyruvate to formate under near-neutral pH. This aligns with pH-dependent transcriptional control seen in other fermentative bacteria, where neutral pH favors *pfl* expression while acidic pH suppresses it (Asanuma et al., 1999). A late metabolic shift toward lactate generation for redox balancing is indicated by upregulated *ldh* and ceasing upregulation of *pflB* on day 8. This temporal pattern supports a model in which pH control stabilizes central carbon entry into PFL while stable hydrogenase maturation cues, leading over time to increased reliance on lactate as an electron sink. When considered as a whole, the observed changes in by-product formation with pH control during fermentation with the organism *T. mirandus* are consistent with the enzyme pH optima described in the literature and the RT-qPCR results in this study. This demonstrates how metabolic pathways can be influenced by pH control toward increased H_2_ synthesis.

Because volatile fatty acid accumulation causes pH to drop during fermentation, only controlled pH conditions allow a clear assessment of pH effects on cellular processes (Jung et al., 2011; Li and Fang, 2007; Łukajtis et al., 2018). Neutral pH values of 7.5 and 8.0 yielded the highest volumetric H_2_ production rates, the fastest lactose consumption, and the shortest lag phase, suggesting an optimum pH range of 7.5–8.0 under the present conditions for *T. mirandus*. It appears that neutral to slightly alkaline pH and high VHPR values are associated with increased formate accumulation, which is consistent with the RT-qPCR observations and literature-reported enzyme pH optima. The observation that *T. mirandus* consumed the full lactose amount and produced similar H_2_ quantities at pH between 6.5 and 8.5 indicates functional limits for H_2_ production across that range, aligning with reported PFL optima on enzymatic activity and gene expression level in organisms that employ similar pathways. Cell proliferation was possible from pH 6.0–8.5. Also *Enterobacter cloacae* has been shown to grow across a broader range, pH 4–11, than the window that enables H_2_ production (pH 4–7) in this organisms (Kumar and Das, 2000). This emphasizes the significance of maintaining optimal pH levels when the aim is to maximize H_2_ production and not biomass formation.

A decline in H_2_ yield with increasing carbon concentration is widely reported for DF (Ferchichi et al., 2005; Łukajtis et al., 2018; Mokhtarani et al., 2025). High substrate concentrations can impose a shock load on cells, causing high initial growth and rapid H_2_ and by-product formation, but leaving residual substrate (Van Ginkel et al., 2001). Metabolic shifts are commonly observed under these conditions (Hallenbeck, 2005; Mokhtarani et al., 2025). In the present case, lactate yields were similar across lactose concentrations, whereas acetate and ethanol declined with increasing lactose concentration. Nevertheless, the highest VHPR between day 4 and day 8 with 13.9 mmol lactose L^−1^ suggested suitability for scale up with fed carbon source to target maximum VHPR.

To sum up, beyond reaffirming known roles of pH and substrate, this study advances the field in three ways. First, it delivers the first systematic fermentation physiology of *T. mirandus*, expanding its substrate scope (including fructose and xylose), delineating nutrient preferences, and defining an actionable operating window for H_2_ production consistent with an FHL-based pathway. Second, it establishes a transferable small-scale workflow to diagnose and mitigate inhibition and implements a two-step closed-flask pH-control method that enabled near-complete lactose conversion with a 4.2-fold H_2_ yield increase. Despite the presence of limitations, such as stepwise pH correction, which approximates but does not replicate continuous control, and the absence of quantified H_2_ partial-pressure thresholds for inhibition, the present study provides a practical screening strategy prior to scale-up. Third, it offers mechanistic insight into pathway partitioning: under pH control, product formation shifted from lactate toward acetate/ethanol and H_2_, and RT-qPCR showed pH-responsive transcription (upregulation of *pflB* on day 4 and *ldh* by day 8), indicating that pH modulates PFL/FHL vs. LDH flux in *T. mirandus*.

## 5 Conclusion

In conclusion, batch cultivations with the newly described *Thermoactinomyces mirandus* as H_2_-producing organism revealed the following key lessons:

- *Controlled pH is essential:* pH 7.2 correction enabled near-complete lactose degradation and a 4.2-fold increase in H_2_ yield, with peak rates at pH 7.5–8.0.
- *Acidification is the main inhibitor*: supplementation of trace elements, Fe^2+^, formate, or N_2_ sparging had minimal effect.
- *RT-qPCR mechanistic insight under pH control:* time-dependent redistribution among PFL/FHL and LDH routes (upregulation of *pflB* on day 4 and *ldh* by day 8).
- *Substrate strategy matters*: higher initial lactose increased volumetric H_2_ rates but lowered yield per hexose equivalent, favoring fed-substrate approaches.
- *Best nutrients*: yeast extract as nitrogen source; fructose, xylose, and lactose as carbon source gave highest H_2_ yields.
- *Energy recovery from by-products:* lactate, acetate, ethanol, and residual formate can be further converted to methane or H_2_ in co-cultures or two-stage processes.
- *Biomass valorization potential*: conversion of lactose, fructose, xylose, and glucose enables H_2_ production from dairy side streams, food waste, and lignocellulosic hydrolysates.
- *C/N ratio modulates metabolism:* for fructose and xylose, higher H_2_ coincided with more acetate and ethanol and less lactate; for lactose, H_2_ scaled with other fermentation products.

These findings advance the fermentation physiology of *T. mirandus* and provide a practical workflow to identify and mitigate inhibitory factors in dark fermentation. The results support bioreactor designs that combine neutral pH control, effective complex nitrogen sources, and gas phase management to sustain high H_2_ productivity. Future work targets scaling up to fed-batch or continuous operation, validating pathway activity through enzyme and omics analyses, and exploring fermentation of real waste streams. These approaches will help transfer the results to industrially relevant conditions and maximize overall energy recovery.

## Data Availability

The raw data supporting the conclusions of this article will be made available by the authors, without undue reservation.
