# Supplementary material for: Optimizing dark fermentation for hydrogen production: lessons from Thermoactinomyces mirandus
Source: Front Microbiol. 2026 Jun 26;17:1827551. doi: 10.3389/fmicb.2026.1827551 (PMC13350025; doi:10.3389/fmicb.2026.1827551)
Supplement: Supplementary file 1 [file Data_Sheet_1.pdf]

Optimizing Dark Fermentation for Hydrogen Production: Lessons from *Thermoactinomyces mirandus*

Supplementary Material

Selina V. Haller[1,2*], Luise Ebert[2], Mira Mutschlechner[2], Harald Schöbel[2] and Andreas O. Wagner[1]

[1]: Universität Innsbruck, Department Microbiology, Innsbruck, Austria

[2]: MCI Internationale Hochschule GmbH, Department Biotechnology, Innsbruck, Austria

*** Correspondence:**
Selina V. Haller
selina.haller@mci.edu

Supplementary Table S1. Volumetric hydrogen production rate VHPR (mean ± SD) of a 8-day *T. mirandus* cultivation with different carbon sources/ substrates in combination with yeast extract. The VHPR is displayed for the specified timeframes, and the maximum values for each carbon source are highlighted in bold font.

Carbon source	VHPR [mmol L^{-1} d^{-1}]<br>0-4 days	VHPR [mmol L^{-1} d^{-1}]<br>4-8 days
Lactose	**0.38 ± 0.08**	0.08 ± 0.11
Glucose	**0.18 ± 0.02**	< 0.01
Fructose	**0.52 ± 0.01**	< 0.01
Xylose	**0.51 ± 0.05**	< 0.01
Arabinose	**0.06 ± 0.02**	< 0.01

Supplementary Table S2. Volumetric substrate consumption rate VSCR (mean ± SD) of a 8-day *T. mirandus* cultivation with different carbon sources/ substrates in combination with yeast extract. The VSCR is displayed for the specified timeframes, and the maximum values for each carbon source are highlighted in bold font.

Carbon source	VSCR [mmol L^{-1} d^{-1}]<br>0-2 days	VSCR [mmol L^{-1} d^{-1}]<br>2-4 days	VSCR [mmol L^{-1} d^{-1}]<br>4-8 days
Lactose	0.11 ± 0.03	**0.70 ± 0.16**	0.15 ± 0.11
Glucose	**0.76 ± 0.19**	0.05 ± 0.07	0.03 ± 0.01
Fructose	**1.03 ± 0.23**	0.25 ± 0.24	0.03 ± 0.24
Xylose	**1.06 ± 0.28**	0.36 ± 0.25	0.29 ± 0.15
Arabinose	**0.38 ± 0.02**	0.28 ± 0.46	0.22 ± 0.13

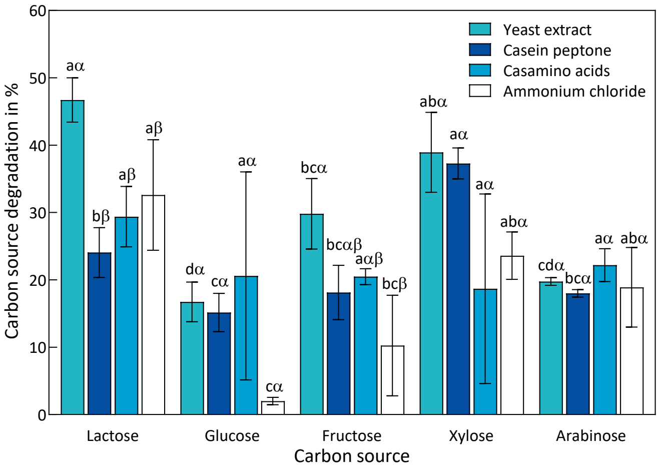

Supplementary Figure S1. Carbon source degradation (mean ± SD) for all combinations of carbon sources (lactose, glucose, fructose, xylose, arabinose) and nitrogen sources (yeast extract, casein peptone, casamino acids, ammonium chloride). Significant differences ($p \leq 0.05$) are indicated by different characters. Lower case characters indicate comparison of carbon sources with equal nitrogen source and Greek characters comparison of nitrogen sources with equal carbon sources.

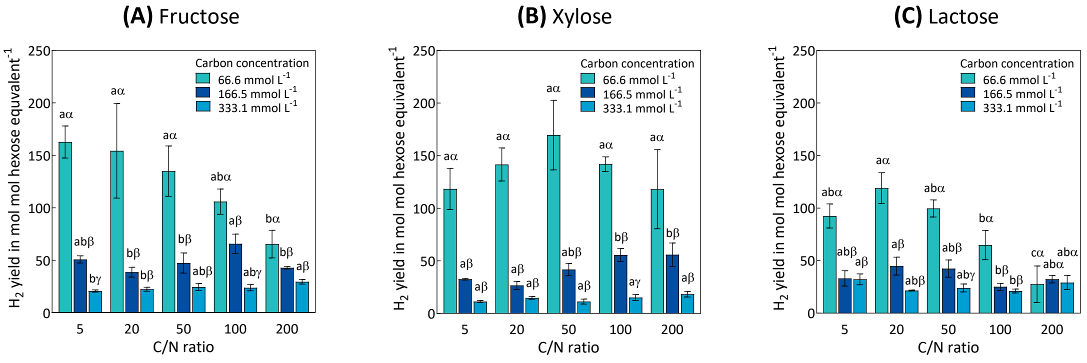

Supplementary Figure S2. Effects of C/N ratio on H_2 yield per hexose equivalent (mean ± SD) during a *T. mirandus* cultivation over 8 days with yeast extract as nitrogen source and three different carbon sources. Significant differences ($p \leq 0.05$) are indicated by different characters. Lower case characters indicate comparison of H_2 yield between equal carbon concentration and Greek characters comparison of H_2 yield between equal C/N ratios. (A) Fructose. (B) Xylose. (C) Lactose.

Supplementary Table S3. Effects of C/N ratio on the volumetric H_2 production rate VHPR (mean ± SD) during a 8-day *T. mirandus* cultivation with different carbon sources (fructose, xylose and lactose) in combination with yeast extract. The data is shown exemplary for the carbon concentration of 66.6 mmol C L^{-1}. The VHPR is displayed for the specified timeframes, and the maximum values for each experiment are highlighted in bold font.

Carbon source	C/N ratio	VHPR [mmol L^{-1} d^{-1}] 0-4 days	VHPR [mmol L^{-1} d^{-1}] 4-8 days
Fructose	5	**0.33 ± 0.05**	0.13 ± 0.08
	20	**0.35 ± 0.02**	0.10 ± 0.12
	50	0.04 ± 0.03	**0.35 ± 0.04**
	100	**0.18 ± 0.04**	0.14 ± 0.09
	200	**0.10 ± 0.06**	0.08 ± 0.09
Xylose	5	**0.28 ± 0.03**	0.03 ± 0.01
	20	**0.20 ± 0.12**	0.19 ± 0.14
	50	0.10 ± 0.06	**0.32 ± 0.13**
	100	0.05 ± 0.01	**0.31 ± 0.01**
	200	0.10 ± 0.08	**0.21 ± 0.01**
Lactose	5	**0.21 ± 0.06**	0.04 ± 0.05
	20	**0.20 ± 0.15**	0.12 ± 0.11
	50	**0.17 ± 0.08**	0.11 ± 0.08
	100	**0.12 ± 0.09**	0.07 ± 0.06
	200	**0.06 ± 0.06**	0.01 ± 0.01

Supplementary Table S4. Effects of C/N ratio on volumetric substrate consumption rate VSCR (mean ± SD) during a 8-day *T. mirandus* cultivation with different carbon sources (fructose, xylose and lactose) in combination with yeast extract. The data is shown exemplary for the carbon concentration of 66.6 mmol C L^{-1}. The VSCR is displayed for the specified timeframes, and the maximum values for each experiment are highlighted in bold font.

Carbon source	C/N ratio	VSCR [mmol L^{-1} d^{-1}] 0-2 days	VSCR [mmol L^{-1} d^{-1}] 2-4 days	VSCR [mmol L^{-1} d^{-1}] 4-8 days
Fructose	5	0.26 ± 0.32	**0.76 ± 0.21**	< 0.01
	20	0.49 ± 0.39	**0.80 ± 0.24**	< 0.01
	50	0.02 ± 0.03	0.40 ± 0.11	**0.53 ± 0.05**
	100	0.05 ± 0.11	**0.85 ± 0.09**	< 0.01
	200	0.06 ± 0.11	**0.55 ± 0.25**	< 0.01
Xylose	5	**0.83 ± 0.34**	0.56 ± 0.36	0.14 ± 0.09
	20	**0.83 ± 0.36**	0.57 ± 0.36	0.28 ± 0.15
	50	**0.58 ± 0.06**	0.28 ± 0.06	0.47 ± 0.10
	100	0.50 ± 0.07	< 0.01	**0.82 ± 0.41**
	200	0.51 ± 0.10	< 0.01	**0.62 ± 0.24**
Lactose	5	0.09 ± 0.18	**0.58 ± 0.26**	0.08 ± 0.05
	20	< 0.01	**0.45 ± 0.24**	0.21 ± 0.13
	50	0.20 ± 0.22	**0.39 ± 0.05**	0.11 ± 0.15
	100	< 0.01	**0.45 ± 0.29**	0.13 ± 0.14
	200	0.04 ± 0.11	**0.25 ± 0.16**	< 0.01

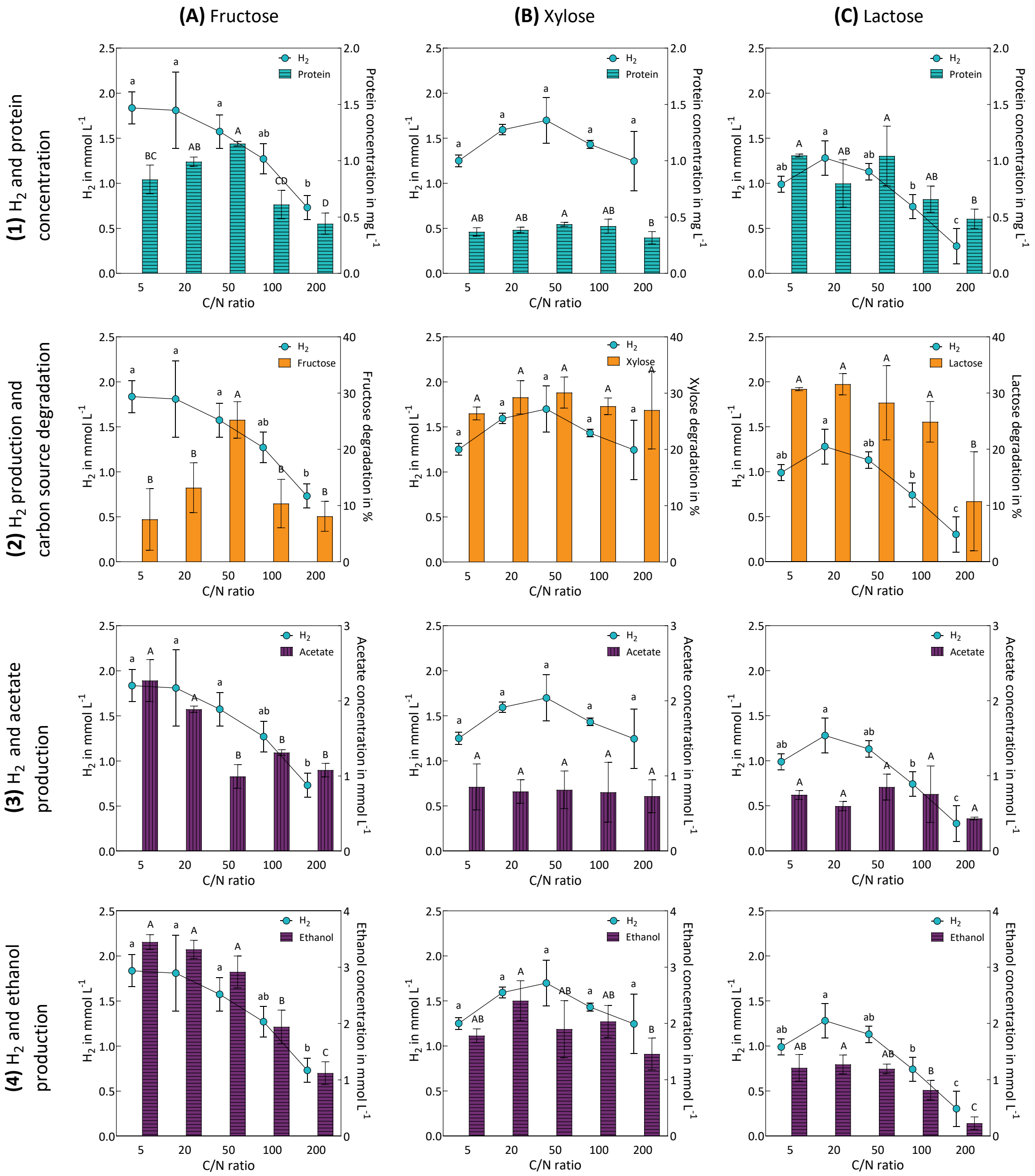

Supplementary Figure S3. Effects of C/N ratio on different fermentation parameters during a *T. mirandus* cultivation over 8 days with yeast extract as nitrogen source, three different carbon source and a carbon concentration of 66.6 mmol C L^{-1}. Significant differences ($p \leq 0.05$) are indicated by different characters. Lower case characters indicate comparison of H_2 production and upper case characters comparison of the additional fermentation parameter. Rows: (A) Fructose. (B) Xylose. (C) Lactose. Columns: (1) Volumetric protein and H_2 production (mean ± SD). (2) Carbon source degradation and volumetric H_2 production (mean ± SD). (3) Volumetric acetate and H_2 production (mean ± SD). (4) Volumetric ethanol and H_2 production (mean ± SD).

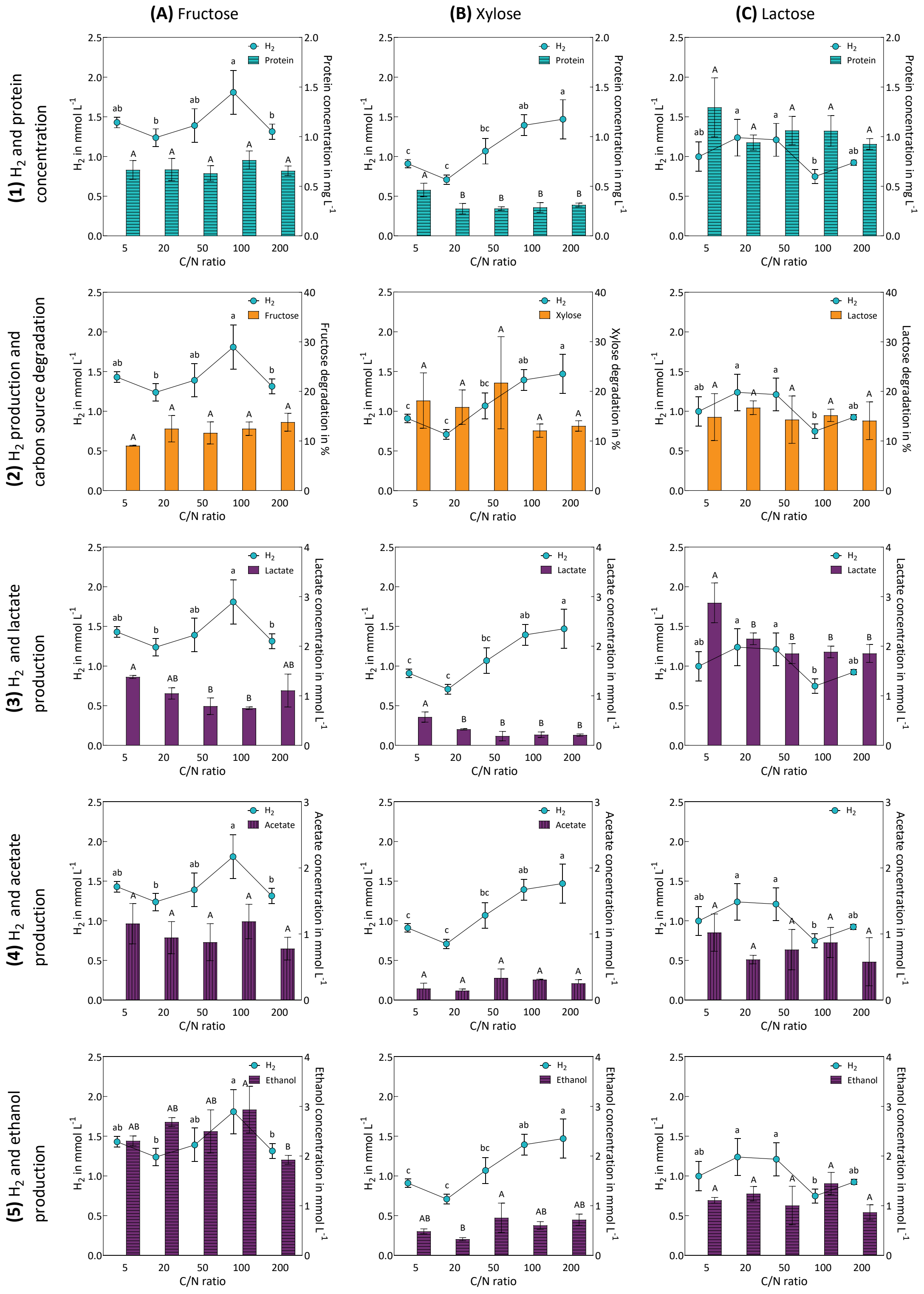

Supplementary Figure S4. Effects of C/N ratio on different fermentation parameters during a *T. mirandus* cultivation over 8 days with yeast extract as nitrogen source, three different carbon source and a carbon concentration of 166.5 mmol C L^{-1}. Significant differences ($p \leq 0.05$) are indicated by different characters. Lower case characters indicate comparison of H_2 production and upper case characters comparison of the additional fermentation parameter. Rows: (A) Fructose. (B) Xylose. (C) Lactose. Columns: (1) Volumetric protein and H_2 production (mean ± SD). (2) Carbon source degradation and volumetric H_2 production (mean ± SD). (3) Volumetric acetate and H_2 production (mean ± SD). (4) Volumetric ethanol and H_2 production (mean ± SD).

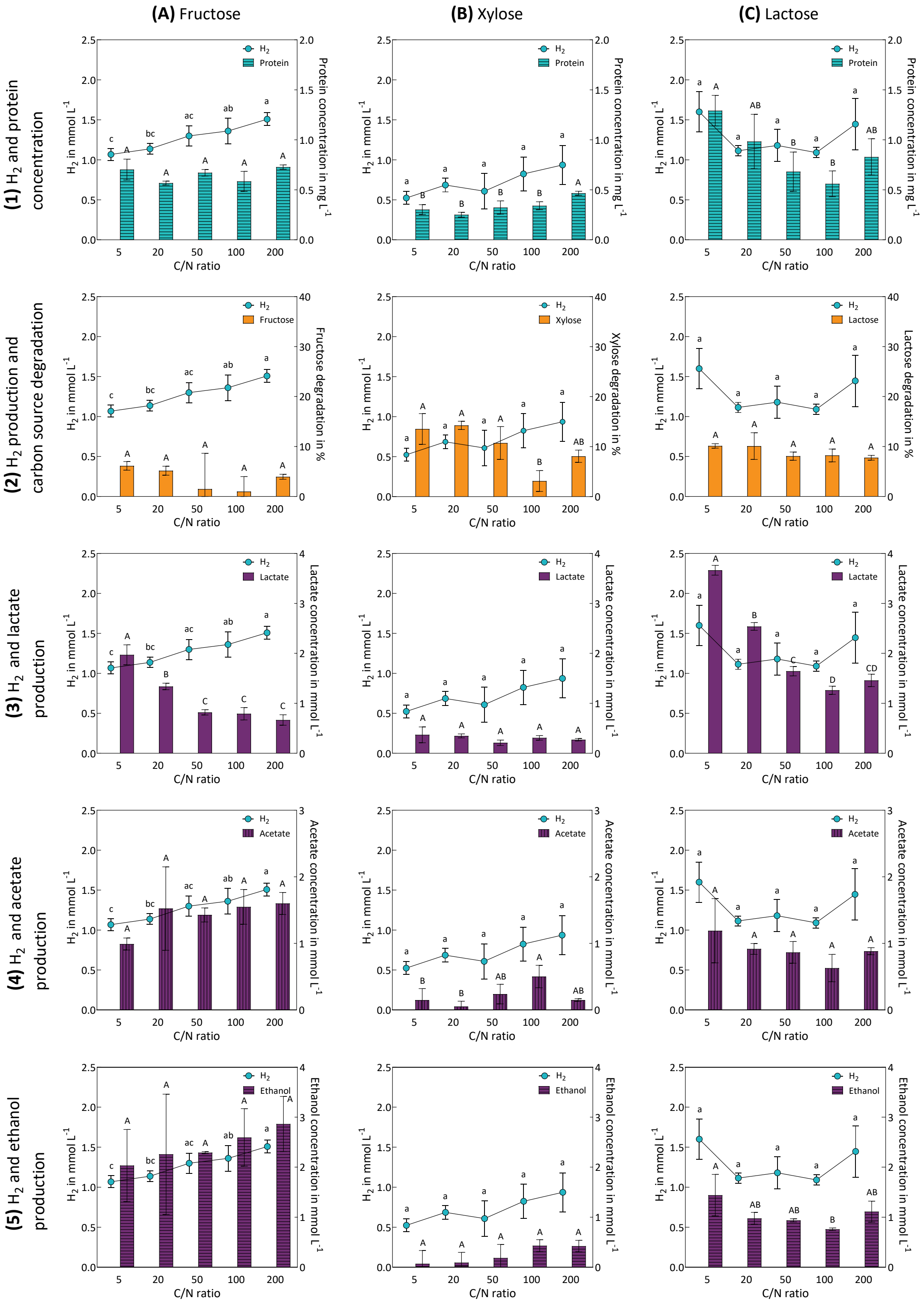

Supplementary Figure S5. Effects of C/N ratio on different fermentation parameters during a *T. mirandus* cultivation over 8 days with yeast extract as nitrogen source, three different carbon source and a carbon concentration of 333.1 mmol C L^{-1}. Significant differences ($p \leq 0.05$) are indicated by different characters. Lower case characters indicate comparison of H_2 production and upper case characters comparison of the additional fermentation parameter. Rows: (A) Fructose. (B) Xylose. (C) Lactose. Columns: (1) Volumetric protein and H_2 production (mean ± SD). (2) Carbon source degradation and volumetric H_2 production (mean ± SD). (3) Volumetric acetate and H_2 production (mean ± SD). (4) Volumetric ethanol and H_2 production (mean ± SD).

Supplementary Table S5. RT-qPCR primer used in this study.

Enzyme	Gene	Primer	Sequence
L-lactate dehydrogenase	*ldh*	Forward	5'-GGCTGTTTACGACGTGATGC-3'
		Reverse	3'-CCACTCGTTAACTTCCGCCT-5'
Pyruvate formate lyase/ formate C-acetyltransferase	*pflB*	Forward	5'-CGGAGGAATCGGTCTGGATG-3'
		Reverse	3'-TACCGTCAGGTTTGGTTCCG-5'
Hydrogenase formation protein HypD	*hypD*	Forward	5'-GGCGGTGAATAGACTACCCG-3'
		Reverse	3'-GACGCCATGATCAAACTCGC-5'
DNA gyrase subunit A	*gyrA*	Forward	5'-CGCATGTCGAGAATTGCCTG-3'
		Reverse	3'-TCTGAACAAGGCCGAGGAAC-5'

Supplementary Table S6. Normalized expression ratio (reference/target; mean ± SD) for a *T. mirandus* batch cultivation without pH control (starting point 7.2) and pH control at setpoint 7.2 over 8 days. The expression of the genes *pflB, hypD* and *ldh* was normalized to the expression of *gyrA.* Significant differences with $p \leq 0.05$ between no pH control and pH control are highlighted in bold font.

Cultivation period [d]	pH control	*pflB*	*hypD*	*ldh*
0	No/Yes	0.08 ± 0.02	0.05 ± 0.03	0.05 ± 0.02
4	No	**0.71 ± 0.07**	0.77 ± 0.10	0.67 ± 0.07
	Yes	**1.00 ± 0.11**	0.77 ± 0.08	0.73 ± 0.08
8	No	0.87 ± 0.11	1.05 ± 0.05	**0.79 ± 0.06**
	Yes	0.82 ± 0.05	1.03 ± 0.06	**0.93 ± 0.06**

Supplementary Table S7. Volumetric H_2 production rate VHPR (mean ± SD) for a *T. mirandus* batch cultivation without pH control (starting point 7.2) and pH control at fixed pH setpoints from 6.0 to 9.0 over 11 days. The VHPR is displayed for the specified timeframes, and the maximum values for each experiment are highlighted in bold font.

pH control setpoint	VHPR [mmol L^{-1} d^{-1}] 0-4 days	VHPR [mmol L^{-1} d^{-1}] 4-8 days	VHPR [mmol L^{-1} d^{-1}] 8-11 days
No pH control	**0.30 ± 0.05**	0.04 ± 0.08	< 0.01
6.0	0.05 ± 0.02	0.15 ± 0.02	**0.38 ± 0.25**
6.5	0.34 ± 0.09	**0.81 ± 0.04**	0.10 ±0.02
7.0	0.50 ± 0.05	**0.67 ± 0.07**	0.07 ± 0.02
7.5	**0.89 ± 0.15**	0.28 ± 0.07	0.01 ± 0.01
8.0	**0.94 ± 0.05**	0.19 ± 0.05	< 0.01
8.5	0.38 ± 0.05	**0.50 ± 0.16**	0.15 ± 0.11
9.0	< 0.01	< 0.01	< 0.01

Supplementary Table S8. Volumetric substrate consumption rate VSCR (mean ± SD) for a *T. mirandus* batch cultivation without pH control (starting point 7.2) and pH control at fixed pH setpoints from 6.0 to 9.0 over 11 days**.** The VSCR is displayed for the specified timeframes, and the maximum values for each experiment are highlighted in bold font.

pH control setpoint	VSCR [mmol L^{-1} d^{-1}] 0-2 days	VSCR [mmol L^{-1} d^{-1}] 2-4 days	VSCR [mmol L^{-1} d^{-1}] 4-8 days	VSCR[mmol L^{-1} d^{-1}] 8-11 days
No pH control	**0.46 ± 0.28**	0.29 ± 0.33	< 0.01	< 0.01
6.0	0.01 ± 0.07	0.11 ± 0.11	0.19 ± 0.04	**0.29 ± 0.14**
6.5	0.03 ± 0.15	**0.82 ± 0.12**	0.63 ± 0.05	0.03 ± 0.02
7.0	0.14 ± 0.05	**1.01 ± 0.09**	0.49 ± 0.05	0.01 ± 0.01
7.5	0.20 ± 0.11	**1.58 ± 0.07**	0.15 ± 0.05	< 0.01
8.0	0.37 ± 0.11	**1.53 ± 0.11**	0.08 ± 0.04	< 0.01
8.5	0.11 ± 0.01	**0.92 ± 0.08**	0.46 ± 0.09	0.06 ± 0.09
9.0	0.12 ± 0.51	**0.66 ± 0.29**	0.13 ± 0.04	0.14 ± 0.04

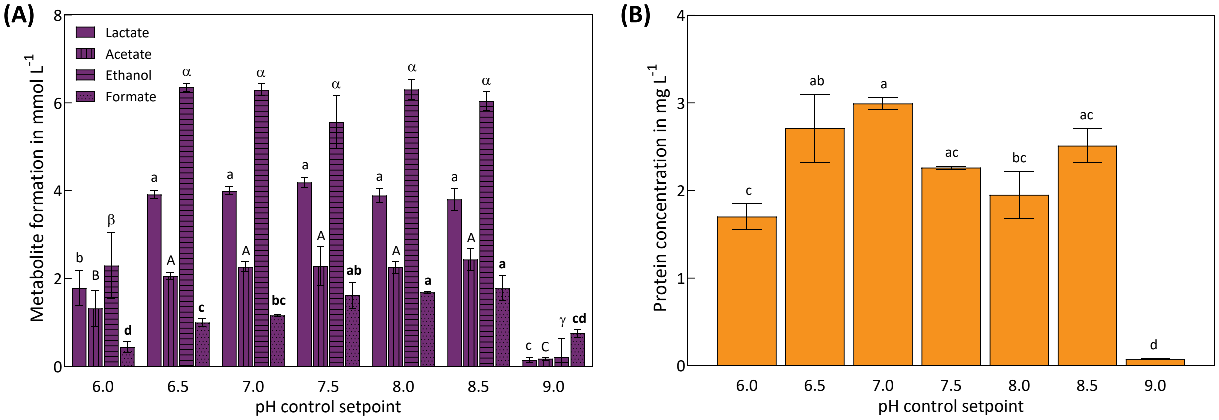

Supplementary Figure S6. Batch fermentations with pH controlled at setpoints from 6.0 to 9.0 over 11 days. (A) End-point metabolite concentrations (mean ± SD). Lower case characters indicate comparison of lactate concentrations, upper case characters comparison of acetate concentrations, Greek characters comparison of ethanol concentrations, and bold lower case characters comparison of formate concentrations. (B) Endpoint protein concentration (mean ± SD). Significant differences ($p \leq 0.05$) are indicated by different characters.

Supplementary Table S9. Volumetric H_2 production rate VHPR (mean ± SD)) of a 11-day *T. mirandus* cultivation with pH control at setpoint 7.2 at different initial lactose concentrations (5.6, 13.9, 27.8 mmol L^{-1}), compared to no pH control at 5.6 mmol L^{-1} (initial pH 7.2). The VHPR is displayed for the specified timeframes, and the maximum values for each experiment are highlighted in bold font.

Lactose [mmol L-1]	pH control	VHPR [mmol L^{-1} d^{-1}] 0-4 days	VHPR [mmol L^{-1} d^{-1}] 4-8 days	VHPR [mmol L^{-1} d^{-1}] 8-11 days
5.6	No	**0.31 ± 0.65**	< 0.01	< 0.01
5.6	Yes	**0.64 ± 0.03**	0.63 ± 0.06	0.02 ± 0.03
13.9	Yes	0.56 ± 0.01	**1.58 ± 0.06**	0.62 ± 0.06
27.8	Yes	0.68 ± 0.09	**1.40 ± 0.09**	0.77 ± 0.16

Supplementary Table S10. Volumetric substrate consumption rate VSCR (mean ± SD) of a 11-day *T. mirandus* cultivation with pH control at setpoint 7.2 at different initial lactose concentrations (5.6, 13.9, 27.8 mmol L^{-1}), compared to no pH control at 5.6 mmol L^{-1} (initial pH 7.2). The VSCR is displayed for the specified timeframes, and the maximum values for each experiment are highlighted in bold font.

Lactose [mmol L-1]	pH control	VSCR [mmol L^{-1} d^{-1}] 0-2 days	VSCR [mmol L^{-1} d^{-1}] 2-4 days	VSCR [mmol L^{-1} d^{-1}] 4-8 days	VSCR[mmol L^{-1} d^{-1}] 8-11 days
5.6	No	**0.46 ± 0.28**	0.29 ± 0.33	< 0.01	< 0.01
5.6	Yes	0.20 ± 0.04	**1.29 ± 0.08**	1.21 ± 0.05	< 0.01
13.9	Yes	0.39 ± 0.07	1.49 ± 0.11	**4.22 ± 0.24**	0.78 ± 0.03
27.8	Yes	0.69 ± 0.11	2.77 ± 0.07	**6.52 ± 0.41**	2.63 ± 0.34
